# Study on Recovery Strategy of Hearing Loss & SGN Regeneration Under Physical Regulation

**DOI:** 10.1002/advs.202410919

**Published:** 2024-12-23

**Authors:** Zhe Li, Yijia Gao, Xingyu Chen, Lei Xu, Zhou Li, Renjie Chai

**Affiliations:** ^1^ Department of Neurology Aerospace Center Hospital School of Life Beijing Institute of Technology Beijing 100081 China; ^2^ Department of Otolaryngology‐Head and Neck Surgery Shandong Provincial ENT Hospital Shandong University Jinan 250022 China; ^3^ Beijing Institute of Nanoenergy and Nanosystems Chinese Academy of Sciences Beijing 101400 China; ^4^ School of Nanoscience and Engineering University of Chinese Academy of Sciences Beijing 100049 China; ^5^ Co‐Innovation Center of Neuroregeneration Nantong University Nantong 226001 China; ^6^ State Key Laboratory of Digital Medical Engineering Department of Otolaryngology Head and Neck Surgery Zhongda Hospital School of Life Sciences and Technology School of Medicine Advanced Institute for Life and Health Jiangsu Province High‐Tech Key Laboratory for Bio‐Medical Research Southeast University Nanjing 210096 China

**Keywords:** regenerations, hearing losses, spiral ganglion neurons, physical regulations

## Abstract

The World Health Organization (WHO) reports that by 2050, nearly 2.5 billion people are expected to have some degree of hearing loss (HL) and at least 700 million will need hearing rehabilitation. Therefore, there is an urgent need to develop treatment strategies for HL. At present, the main treatment strategies for HL are hearing aids and cochlear implants (CIs), which cannot achieve a radical cure for HL. Relevant studies have shown that the most fundamental treatment strategy for sensorineural hearing loss (SNHL) is to regenerate hair cells and spiral ganglion neurons (SGNs) through stem cells to repair the structure and function of cochlea. In addition, physical stimulation strategies, such as electricity, light, and magnetism have also been used to promote SGN regeneration. This review systematically introduces the classification, principle and latest progress of the existing hearing treatment strategies and summarizes the advantages and disadvantages of each strategy. The research progress of physical regulation mechanism is discussed in detail. Finally, the problems in HL repair strategies are summarized and the future development direction is prospected, which could provide new ideas and technologies for the optimization of hearing treatment strategies and the research of SGN repair and regeneration through physical regulation.

## Graphical abstract

This paper systematically summarizes the development of existing hearing loss (HL) recovery strategies and the latest research of spiralganglion neurons (SGNs) regeneration under physical regulation. The challenges facing physically regulating SGNs are discussed, and the future development are prospected. This work will provide a new strategy and method for regulating the life activity of SGNs and hearing recovery.

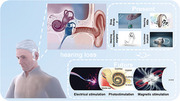

## 1 Introduction

Hearing, as an important sense of access to external information, can make us perceive the environment and generate a sense of security and participation, which plays a key role in the communication of human society. However, with the aging of the population, the abuse of ototoxic drugs, noise and environmental pollution, vestibular schwannoma, and other factors, the number of deafness and hearing loss has gradually increased and has become a global health problem.^[^ ^1^ ^]^ According to the latest data released by World Health Organization (WHO), disabled hearing loss has exceeded 5% (360 million) of the world's population, 32 million of which are children, and ≈1/3 of the elderly over 65 years old have disabled hearing loss (HL).^[^ ^2^ ^]^ According to the location of the lesion, hearing loss can be divided into conductive hearing loss (CHL), sensorineural hearing loss (SNHL), and mixed hearing loss. CHL is caused by the structural and functional disorders of the outer ear and middle ear, which leads to the weakening of the sound introduced into the inner ear. About 63% of the deaf population is SNHL. Sensorineural deafness refers to the lesions of hair cells (HCs), auditory nerves, auditory conduction pathways, or hearing centers at all levels in the cochlea, and the impairment of sound perception and nerve impulse conduction.^[^ ^3^ ^]^ It is a common disease in clinical practice with hearing impairment, HL, or even disappearance accompanied by tinnitus and ear blockage. It is currently believed that mammalian HCs and spiralganglion neurons (SGNs) cannot regenerate spontaneously once damaged, resulting in permanent deafness.^[^ ^4^ ^]^ In addition, hidden hearing loss (HHL) is a recently studied type of acoustic neuropathy.^[^ ^5^ ^]^ Animals and humans with HHL have normal hearing thresholds. However, there is a defect in cochlear neurotransmission, a decrease in the suprathreshold amplitude of sound‐induced auditory nerve complex action potentials. Notably, a benign Schwann cell (SCs) ‐derived acoustic nerve tumor, called vestibular schwannoma (VS), has also been suggested as a cause of HL. Recent research has shown that the transient loss of cochlear Schwann cells also leads to permanent hearing deficits characterized by HHL.^[^ ^6^ ^]^

Common clinical treatments for deafness include drug therapy,^[^ ^7^ ^]^ hearing aids,^[^ ^8^, ^9^ ^]^ hearing reconstruction,^[^ ^10^, ^11^ ^]^ and cochlear implants (CIs).^[^ ^12^, ^13^ ^]^ Although these methods can improve patients' hearing to a certain extent, their effectiveness depends entirely on the number and quality of HCs and SGN remaining in the patient's inner ear, which cannot fundamentally cure deafness. Therefore, it is necessary to develop a fundamental treatment for deafness. At present, the most fundamental treatment for sensorineural deafness is to promote the proliferation and differentiation of inner ear stem cells, and regenerate HCs and SGN, so as to achieve the repair of the structure and function of the cochlea. In addition, glial cell (i.e., glia‐like support cells, SCs, or satellite cells) reprogramming to HC or SGN is also an alternative approach that deserves continued research in the future.^[^ ^1^ ^]^ Researchers found that Sox2 + subpopulation of cochlear glial cells preserves the high potency of neuronal differentiation. A combination of small molecules not only promoted neuronal differentiation of Sox2 − glial cells but also removed glial cell identity and promoted the maturation of the induced neurons (iNs) toward SGN fate.^[^ ^14^ ^]^ However, even if this method can obtain regenerated hair cells and SGN, it is necessary for the regenerated spiral ganglion neuron neurites to grow and distribute to functional hair cells in order to truly repair hearing loss.^[^ ^15^ ^]^ Therefore, repairing and regenerating SGN after injury and loss, and promoting the growth of neurites to make them functional have become the focus of research in the field of hearing in recent years. Physical regulatory factors refer to the factors that regulate organisms or biological systems by physical means.^[^ ^16^, ^17^ ^]^ It mainly includes stimulation, magnetic stimulation, light stimulation, ultrasonic stimulation, temperature regulation, mechanical stimulation, and so on. These physical regulatory factors are widely used in biomedical engineering and can be used in diagnosis,^[^ ^18^ ^]^ treatment,^[^ ^19^ ^]^ rehabilitation,^[^ ^20^ ^]^ and disease prevention.^[^ ^21^ ^]^ Among them, electrical stimulation is to stimulate organisms or biological tissues by applying an electric current, which is often used in the treatment of nervous system diseases,^[^ ^22^ ^]^ rehabilitation treatment,^[^ ^23^ ^]^ cardiac pacing,^[^ ^24^ ^]^ and other fields. Magnetic stimulation is a kind of technical means to use the influence of magnetic field (MF) on the organism for nerve regulation,^[^ ^25^, ^26^ ^]^ bone growth stimulation,^[^ ^27^, ^28^ ^]^ tumor treatment,^[^ ^29^, ^30^, ^31^ ^]^ and so on. Photostimulation is the use of light to affect organisms and has full applications in dermatological therapy,^[^ ^32^, ^33^ ^]^ optogenetics,^[^ ^34^, ^35^ ^]^ or photodynamic therapy.^[^ ^36^, ^37^ ^]^ With the continuous development of technology, the application field of physical regulatory factors is still expanding. Physical regulation has also been widely studied and proved to be effective in the repair and regeneration of SGN after injury and loss, as well as the growth of neurite of SGN. For example, electrical stimulation has been shown to promote regeneration of SGNs,^[^ ^38^, ^39^ ^]^ promote stem cell regeneration of SGNs,^[^ ^40^, ^41^ ^]^ protect SGNs,^[^ ^42^, ^43^ ^]^ etc. Infrared nerve stimulation has been used to repair cochlear injury and regulate the electrical activity of auditory neurons in animal models.^[^ ^44^, ^45^ ^]^ The optical cochlear implant based on photogenetics can effectively stimulate the SGN in the cochlea.^[^ ^46^, ^47^ ^]^ In addition, the regeneration and neural regulation of SGNs can also be achieved by direct uptake of SPION and the construction of a biomimetic extracellular matrix with magnetic response.^[^ ^48^, ^49^ ^]^ Although a number of studies have demonstrated the promoting effect of physical regulation on SGNs repair and regeneration, the specific molecular mechanisms of various regulations still need to be further clarified.

**Figure 1:**
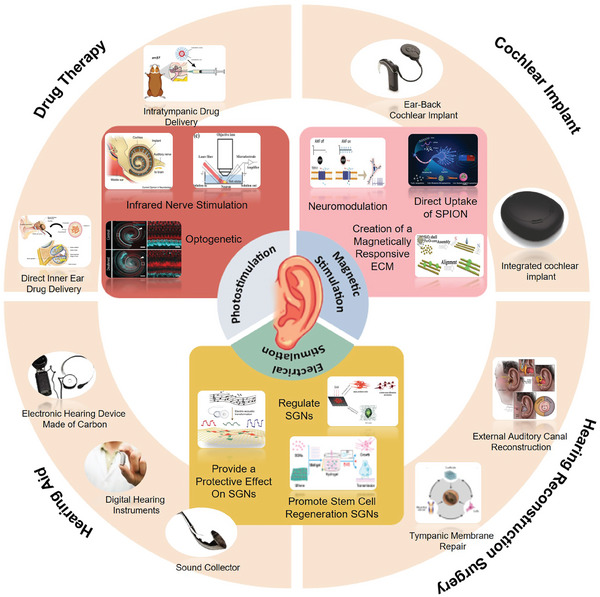
Schematic classification of hearing treatment strategies and physical regulation strategies.

This paper systematically discusses the classification, principle, development, advantages, and disadvantages of the existing hearing loss recovery strategies. It summarizes and discusses the mechanism of physical regulation in the field of SGNs repair and regeneration, and the latest research progress (**Figure** **1**). Finally, the challenges facing physically regulating SGNs repair and regeneration are summarized, and the future development prospects are prospected. This work will provide a new strategy and method for regulating the life activity of SGN through physical factors in clinical application and lay a theoretical foundation for hearing recovery and regeneration.

## 2 Existing Treatment Strategies for HL

### 2.1 Drug Therapy

Drug therapy is one of the most important strategies for the treatment of hearing loss, which mainly utilizes drugs to target the relevant sites of sensorineural hearing loss to achieve therapeutic goals. Drug therapy strategies are categorized according to the route of administration, including systemic and local drug delivery. Local drug delivery is further divided into intratympanic drug delivery (middle ear drug delivery) and direct inner ear drug delivery.

#### 2.1.1 Systemic Drug Delivery

Systemic drug delivery in combination with steroids, vasodilators, neurotrophic factors, thrombolytics, or hyperbaric oxygen (HBO) is commonly used in the treatment of SNHL. However, after systemic administration, it is difficult to achieve effective therapeutic concentrations because of the obstruction of the blood‐labyrinth barrier (BLB), which makes it difficult for the drug to be effectively delivered to the target site in the inner ear. Therefore, systemic drug delivery has gradually been replaced by local drug delivery or a combination of systemic and local drug delivery. For example, Yang^50^et al. compared the pharmacokinetic differences between combination therapy (CT), intravenous therapy (IV) alone, and transtympanic therapy (TT) in the ears of transgenic GFAP‐Luc mice using a protocol for assessing D‐fluorescein distribution. The in vivo imaging system (IVIS) showed significantly increased biological half‐life, total photon count, and area under the curve (AUC) values in the CT group. This study suggests that CT has enhanced drug delivery capabilities and is more clinically useful compared to TT and IV.

#### 2.1.2 Local Drug Delivery

##### Intratympanic Drug Delivery

Intratympanic drug delivery is a method to achieve the therapeutic effect by injecting or perfusing the drug preparation in the form of solution, suspension, or injectable gel into the middle ear. The drug enters the corresponding target of action in the inner ear through the round window membrane (RWM) or other regions of the inner ear. Compared with systemic drug delivery, intratympanic drug delivery bypasses the vagal artery and the blood‐vague barrier,^[^ ^51^ ^]^ which is a more direct and effective route of drug delivery. At present, the research focus of this method of drug delivery mainly includes the biosafety of the drug preparation and the contact time of the drug with the round window film.

Since locally administered preparations in the middle or inner ear lead to the cerebrospinal fluid via the external lymphatic fluid, it can cause damage to the central nervous system with the preservatives in the preparation. In addition, the presence of preservatives in the middle ear will affect the permeability of the round window membrane.^[^ ^52^ ^]^ And when these preservative molecules are concentrated in the inner ear, ototoxicity may be produced.^[^ ^53^ ^]^ Based on this, there is the disallowance of preservatives in topically administered formulations for the middle or inner ear. Finally, excipients must be biosafe, which is required to be hypoimmunogenic and minimize inflammatory responses. Therefore, based on biocompatibility considerations, higher demands are placed on drug delivery formulations for intracochlear administration.

The key parameter that affects the drug concentration in the exolymph after intra‐drum administration is the contact time between the drug and the round window membrane. In order to increase the drug concentration in the exolymph and reduce the variability of its drug level, the residence time of the drug at the round window membrane should be maintained and controlled as much as possible after the drug comes into contact with the membrane. However, drug solutions or suspensions, such as gentamicin solution and dexamethasone solution, are commonly used in clinical practice for intra‐tubular administration of drugs to treat the inner ear disorders. These drug formulations have a short residence time in the middle ear due to the clearing action of the Eustachian tube, thereby shortening the contact time with the round window membrane. As a consequence, developing strategies for long‐term contact between drugs and round window film has been one of the research focuses of inner ear drug delivery.

In order to construct a more effective and safe drug delivery system, it is essential to prolong the retention time of drugs in the middle ear and increase their contact time with the round window membrane. Therefore, two effective drug delivery systems have been developed: implantable medical drug delivery systems and injectable drug delivery systems.^[^ ^54^ ^]^

Implantable medical drug delivery systems utilize microcores, microcatheters, or biodegradable/non‐biodegradable polymer devices. A common feature of these devices is that they allow drug delivery from the outer ear to the surface of the round window membrane, thereby increasing the contact time with the membrane. Zou et al. slowly released liposome nanoparticles that could be loaded with therapeutic drugs for inner ear disorders into the tympanic chamber via a micro‐osmotic pump, followed by an in vivo uptake study by magnetic resonance imaging (MRI).^[^ ^55^ ^]^ The results showed that liposomes could enter the inner ear after release by this micro‐osmotic pump without causing adverse effects. In a clinical trial, Plontke et al. verified the safety and efficacy of continuous intratympanic dexamethasone injection through a round window catheter for the treatment of Sudden SNHL by designing a randomized, double‐blind treatment group and placebo‐controlled trial.^[^ ^6^ ^]^ The mean change in 4 PTA was 13.9 dB and 5.4 dB in the treatment and placebo groups, respectively. The treatment group showed a significant trend toward hearing improvement.

**Figure 2:**
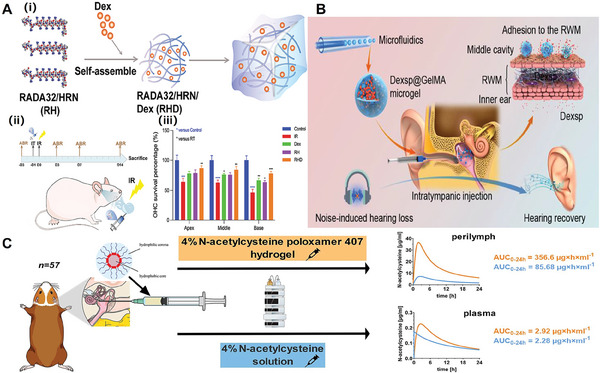
Active carriers in the injectable delivery system. A) i) Pathway for the synthesis of RHD hydrogel.^[^ ^52^ ^]^ ii) Animal experiment program to assess the efficacy of RHD hydrogel for the prevention of IR‐induced hearing loss in vivo. iii) A comparison of outer hair cell survival between experimental groups. Reproduced with permission. Copyright 2023, Elsevier B.V. B) A schematic diagram of Dexsp@GelMA microgel intra‐canal injection for the treatment of NIHL.^[^ ^4^ ^]^ Reproduced with permission. Copyright 2022, American Chemical Society. C) Protocol for injection of Poroxamer 407 hydrogel within the tympanic chamber for continuous delivery of N‐acetylcysteine to the inner ear.^[^ ^53^ ^]^ Reproduced with permission. Copyright 2020, Elsevier B.V. All rights reserved.

In the injectable drug delivery systems, there are some effective carriers such as hydrogels, nanoparticles, and hyper pellets. These carriers have been used to treat inner ear disorders.^[^ ^7^, ^56^, ^57^ ^]^ By designing hydrogel materials with high adhesive properties, the hydrogel can prolong the time of contact with the round window membrane, thereby increasing the concentration of the drug‐loaded therein into the inner ear. As shown in **Figure** **2**A, Liu et al. designed a new injectable hydrogel called RADA32‐HRN‐ dexamethasone (RHD) for the treatment of radiation hearing loss.^[^ ^56^ ^]^ The nanofiber network in this hydrogel gave a great slow‐release effect for the loaded dexamethasone. In an in vivo test in mice, different drugs (PBS, free Dex (Dexamethasone) solution, RH hydrogel, RHD hydrogel) were injected near the niches of the round window of the mice and the ability of each group to protect the hearing function of the mice were evaluated by ABR tests (the auditory brainstem response). The results showed that the RHD hydrogel had a significant protective effect on the hearing function of mice. Wang et al. prepared gelatin‐encapsulated gelatin‐methacryloyl microgel particles encapsulating dexamethasone sodium phosphate (Dexsp@GelMA) (Figure 2B).^[^ ^7^ ^]^ This microgel has a uniform size and adheres tightly to the round window membrane, resulting in a significantly longer contact time of the drug with the round window membrane. The microgel has demonstrated superior efficacy in the treatment of noise‐induced hearing loss (NIHL). In addition, Poroxamer 407 hydrogels (POX407) are a class of hydrogels with reversible thermo‐responsiveness. When the external temperature is close to the body temperature, it rapidly gels and becomes immobilized at the site of implantation. This property makes it ideally suited for drug delivery, where the drug carried is released steadily and continuously at a specific site in the body. Thus, by injecting Poroxamer 407 hydrogel within the tympanic cavity, the researchers can steadily deliver N‐acetylcysteine into the inner ear for the treatment of inner ear diseases. (Figure 2C).^[^ ^57^ ^]^

##### Direct Inner Ear Drug Delivery

Increasing the concentration of the drug in the inner ear region is the main objective of local drug delivery. However, intratympanic administration requires the drug to pass through the RWM barrier that restricts the entry of substances into the inner ear, which also makes direct inner ear administration more effective than intratympanic administration.^[^ ^58^, ^59^, ^60^ ^]^

Direct inner ear administration includes cochlear and vestibular administration (semicircular canal injection). They are both characterized by the ability to effectively increase drug concentrations, smaller doses, and higher drug targeting. It cannot be ignored that these highly invasive modes of drug delivery carry the risk of postoperative complications. This also makes it difficult to advance direct inner ear drug delivery in clinical practice, and even the effectiveness of vestibular drug delivery can only be verified in animal models at present. Therefore, direct inner ear drug delivery places higher demands on surgical operation and drug biocompatibility.

**Figure 3:**
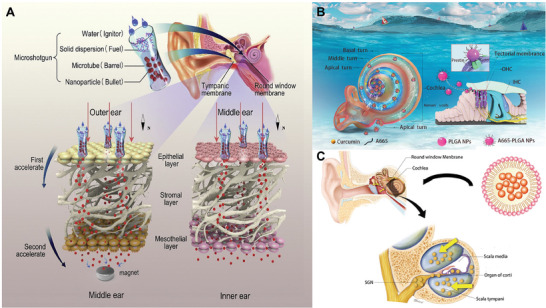
Nanosystems for inner ear drug delivery. A) Mini‐gun delivery system for non‐invasive delivery of MNPs via the tympanic membrane and via the RWM.^[^ ^57^ ^]^ Reproduced with permission. Copyright 2020, Elsevier B.V. All rights reserved. B) A665‐PLGA NPs targeted peptide‐guided delivery system for sufficient delivery to the cochlear tricuspid of the cochlea.^[^ ^58^ ^]^ Reproduced with permission. Copyright 2023, Elsevier B.V. C) Oxidized phospholipid‐based nanoparticles containing dexamethasone optimized for the therapeutic treatment of inner ear disorders.^[^ ^59^ ^]^ Reproduced with permission. Copyright 2018, Elsevier Ltd.

To reduce the risks associated with the highly invasive nature of direct inner ear drug delivery, emerging nanoparticles (NPs) have become the main biomaterials for direct inner ear drug delivery.^[^ ^61^, ^62^, ^63^ ^]^ NPs, which usually have diameters ranging from tens to hundreds of nanometers, can be prepared from different biomaterials and used to encapsulate and deliver a variety of therapeutic drug types. Therapeutic drugs delivered by nanoparticles have higher solubility and stability. At the same time, nanoparticles can control drug release. The above properties enable the therapeutic drugs delivered by NPs to work successfully in the inner ear. Compared with other types of drug delivery materials (e.g., block hydrogels, microcapsules, and microparticles), NPs exhibit higher biocompatibility, targeting, and controllability in the treatment of inner ear diseases, which can effectively improve drug stability. It can also be surface‐modified to improve the release rate, targeting, and other aspects of the drug. Based on these advantages, NPs delivery systems show great potential in the treatment of hearing loss. Researchers have developed a number of nanosystems for inner ear drug delivery, including polymers,^[^ ^64^, ^65^ ^]^ lipid NPs,^[^ ^63^, ^66^ ^]^ superparamagnetic iron oxide NPs,^[^ ^67^ ^]^silica NPs,^[^ ^68^ ^]^ and nanogels.^[^ ^69^ ^]^ Wise^[^ ^70^ ^]^ et al. developed a drug carrier based on superparticles (SPs) constructed from mesoporous silica nanoparticles (≈500 µm). SPs were demonstrated to have the ability to load BDNF efficiently. In in vivo experiments, brain‐derived neurotrophic factor (BDNF)—loaded SPs were surgically implanted into the unilateral cochlea of severely deaf guinea pigs. The SPs were highly biocompatible and provided therapeutic levels of BDNF compared to the contralateral control cochlea without SP implantation, thus significantly improving SGN survival. Thereafter, Nadeschda et al.used drug delivery vehicles based on amino‐modified nanoporous silica nanoparticles (NPSNPs) as a coating for Cis.^[^ ^68^ ^]^ The nanoporous silica nanoparticles were able to achieve long‐term delivery (80 days) of BDNF in an in vitro culture model and improve the in vitro survival of SGNs. The results showed that by combining with a cochlear implant, this drug delivery system could effectively guide the growth of neural synapses toward the cochlear electrode. Liang et al. designed a micro‐shotgun to deliver MNPs through the round window membrane to the inner ear.^[^ ^57^ ^]^ This method uses chemical driving force and external MF force as power sources to drive the loaded MNPs to penetrate the round window membrane efficiently (**Figure** **3**A). The histologic analysis found that the micro‐shotgun could cause little damage to the round window membrane. The employed MNPs were highly biocompatible and did not cause ototoxicity to the experimental guinea pigs. As shown in Figure 3B, Wang et al. employed a strategy of incorporating the targeting peptide A665 to enhance the targeting of drug delivery by poly (lactic‐co‐glycolic acid) nanoparticles (PLGA NPs).^[^ ^62^ ^]^ The targeting peptide A665 binds specifically to prestin which is a protein uniquely expressed in outer hair cells. As a result, A665‐PLGA NPs carrying the antiototoxic drug curcumin can be specifically distributed in the outer hair cell region, increasing the effective concentration of the drug. This approach overcomes the challenge of the difficulty of reaching the apical turn of the cochlea against the flow direction of the drug and is expected to be a new strategy for efficient drug delivery in the inner ear. Moreover, the optimized phospholipid‐based nanoparticles loaded with dexamethasone were applied for the treatment of inner ear disease (Figure 3C).^[^ ^63^ ^]^

Although local drug delivery is one of the most important therapeutic directions for the treatment of inner ear diseases in the future, there are still many aspects that need to be further investigated, and there are also challenges in applying it to the clinic. Therefore, we compared and summarized the advantages and limitations of the two modes of local drug delivery, including invasiveness, applicable materials, efficacy, etc. We hope that this will help in the selection of delivery modes in the clinical setting as well as the exploration of more effective modes of drug delivery in research (**Table** **1**).

**Table 1:** Comparisons of intratympanic and direct inner ear drug delivery.

	Intratympanic drug delivery	Direct inner ear drug delivery
Advantages	Targeted Middle Ear and Inner Ear Disease Treatment	Targeted Inner Ear Disease Treatment
	Drugs do not enter systemic circulation	Drugs do not enter systemic circulation
	Higher concentration of drug reaching the inner ear	The drug bypasses the round window membrane barrier and reaches the inner ear in relatively high concentrations
	High number of clinical practices and more refined techniques	Some carrier materials can be used as cochlear coatings in association with CIs.
	Minimally invasive	Research on gene therapy based on vectors such as AAV can be carried out
	Suitable for hydrogels, implantable medical devices, nanoparticles	Suitable for hydrogels, implantable medical devices, nanoparticles
		Reducing the basal‐apical gradient of drugs in the cochlea
Limitation	High biocompatibility requirements for delivery materials, risk of carrying infectious agents into the middle ear	Highly invasive with risk of postoperative complications
	Inadequate pharmacokinetic studies on drug access to the inner ear through the round window membrane	Clinical practice is scarce, and the technology is not yet perfect
	Round window film properties vary greatly from one individual to another	High concentrations of the drug may lead to potential toxicity
	Difficulty in achieving slow controlled release of drugs	
	Easy clearance of drugs through the eustachian tube space	

### 2.2 Hearing AIDs

There has been rapid development of technology regarding hearing aids. It has become an important tool in hearing rehabilitation^[^ ^9^ ^]^ At present, the primary clinical intervention for people with mild to moderate hearing loss is hearing aids. It can transfer the sound frequency sensed by damaged hair cells to the functional hair cell sensing frequency band by compression frequency shift technology. It can improve hearing loss by amplifying speech sounds and increasing participation in daily life.

Hearing aids are mainly composed of microphones, computing chips, receivers, batteries, various volume, tone control knobs, and other electrical and acoustic devices.^[^ ^71^ ^]^ The microphone in hearing aids is a miniature microphone, which is the main device to helps the hearing‐impaired person to listen. It can pick up all kinds of sounds in the environment and transform them into electrical signals that the processor can recognize. Currently, directional microphones are commonly used in digital hearing aids.^[^ ^71^ ^]^ They can change their polarity according to changes in the surrounding environment automatically, suppress noise, and improve speech intelligibility in noisy environments. However, its disadvantages are that it is susceptible to moisture and clogging.

The hearing aids computing chip is the “heart” of the hearing aids. It includes filters, amplifiers, algorithms, and digital signal‐processing techniques. The filter can divide hearing aids’ audio signal, extract the useful components, and suppress the interfering components in signals.^[^ ^72^ ^]^ Traditional amplifiers simply amplify the signal. The digital hearing instrument amplifiers at present can be customized for different types of deafness and produce a more comfortable and precise hearing aid effectiveness with precision technologies such as maximum output control, automatic gain control, and wide dynamic range compression (WDRC).

Another important device is the receiver, which can convert an amplified electrical signal into an acoustic signal. It utilizes the law of electromagnetic induction to generate magnetic force when the current passes through the internal coil of the receiver. The magnetic force drives the diaphragm to vibrate and produce sound, and the resulting sound signal is then output to the ear canal through the earhook, sound transmission tube, and eardrum.

The principle of hearing aids is acoustoelectric conversion. It can amplify different frequencies of sound according to different hearing loss conditions. First, the sound signal is converted into an electrical signal by a microphone. The weak electrical signal is then amplified by a filter amplifier and processed by various digital signals. Finally, it is restored as a sound signal by the receiver and transmitted to the human ear. The bone‐conduction hearing aids are generally used in special cases, such as congenital aural atresia or aural stenosis, chronic otitis media, and so on.

Currently, hearing aids can be categorized into two main types air‐conduction hearing aids and bone‐conduction hearing aids. The air‐conduction hearing aids are mainstream.^[^ ^9^ ^]^ The bone‐conduction hearing aids are generally used in special cases, such as congenital aural atresia or aural stenosis, chronic otitis media, and so on.

Air conduction hearing aids are transmitted through the air, which can enhance the vibration of the sound in the air of the outer ear canal so that the sound is transmitted to the inner ear through the outer ear and the middle ear, and stimulates the functional hair cells to transmit the signal to the hearing center through the nerve. These devices are intended for people with mild or moderate hearing impairment. Air conduction hearing aids can be divided into behind‐the‐ear (BTE) hearing aids, ear canal hearing aids, ear mold hearing aids, deep ear canal hearing aids, external receiver hearing aids, hidden hearing aids, etc. The current mainstream type is the BTE hearing aid. The device is suspended at the base of the upper edge of the auricle, and the earpiece and earplug are inserted into the opening of the ear canal. It has the advantages of low noise, low distortion, high power, stable performance, simple operation, and easy maintenance.

Bone‐conduction hearing aids can transmit sound signals to the skull and then to the inner ear directly, over the damaged outer or middle ear. According to the different wearing positions, it can be divided into head‐worn bone conduction hearing aids, dental bone conduction hearing aids, and glasses bone conduction hearing aids. Bone‐conduction hearing aids break through the limitations of air‐conduction hearing aids that rely on the outer ear and the middle ear. They are suitable for patients with chronic otitis media, chronic external otitis, and congenital deafness.^[^ ^73^ ^]^ However, its maximum output is smaller than that of air‐conduction hearing aids, and it is inconvenient to wear. Therefore, the choice of two hearing aids needs to be based on the actual situation of the patient.

**Figure 4:**
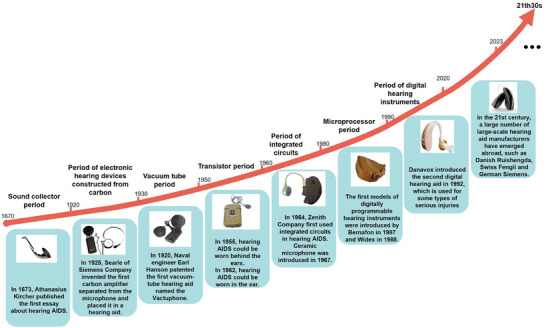
Development history of Hearing AIDs.

Hearing aids are the most common treatment for people with age‐related hearing loss (**Figure** **4**). Hearing aids help patients hear in complex everyday communication environments while ensuring their comfort. However, the use of hearing aids is low. This may be related to social context, social pressure,^[^ ^74^ ^]^ the cost of the device itself, stigma, and lack of cognition among hearing aid users.^[^ ^75^ ^]^ To address this issue, the U.S. Food and Drug Administration (FDA) has approved a new over‐the‐counter hearing aid (OTC).^[^ ^76^ ^]^ At the same time, studies have shown that remote audiology services can improve patient access and reduce care costs and time.^[^ ^77^ ^]^ Therefore, future research should focus on the economy, universality, and comfort of hearing aids.

### 2.3 Hearing Reconstructive Surgery

Hearing reconstruction aims to restore hearing loss by surgical methods, especially to deal with the dysfunction of sound transmission structure caused by different reasons or diseases.^[^ ^78^ ^]^ It is widely used in conductive deafness. A smooth external auditory canal, normal tympanic membrane, complete and active ossicular chain, normal round window membrane, oval window membrane, and smooth eustachian tube are the main conditions to ensure normal sound transmission. According to these factors, hearing reconstruction surgery can be divided into external auditory canal reconstruction, tympanic membrane repair, ossicular chain reconstruction, stapes surgery, and fenestration of the inner ear. In the following, tympanic membrane repair, stapes surgery, and fenestration of the inner ear are briefly introduced.

#### 2.3.1 Tympanic Membrane Repair

Tympanic membrane is a thin film located between the tympanic cavity and the external auditory canal, which separates the external auditory canal from the tympanic cavity. It is the only way for sound waves to enter the cochlea from the external ear. Therefore, the integrity of the tympanic membrane plays an important role in the formation of hearing. When the tympanic membrane is perforated due to external factors, the patients may have hearing deterioration or even loss. Tympanic membrane repair can restore the anatomical structure and the sound wave transmission function of the tympanic membrane, seal the middle ear cavity, and reduce the infection of the middle ear. Although tympanic membrane perforation can repair itself, due to the lack of intermediate fiber, it is very fragile and easy to be perforated twice, and the sound transmission performance is poor. Therefore, it is necessary to reconstruct the three‐layer structure of the eardrum.^[^ ^79^ ^]^ This surgery focuses on repairing the perforated eardrum and does not require treatment of other areas. It only needs to cover the perforation site with a suitable material, and the original cells can migrate on the material by using the scaffold function of the material to promote the healing of the tympanic membrane. Traditionally, the most commonly used material is temporalis fascia, which is easy to obtain, simple to prepare, similar in the thickness to tympanic membrane, and has a high closure rate during surgery.^[^ ^80^ ^]^ At the same time, autologous transplantation of perichondrium is also one of the typical materials for tympanic membrane repair. Due to the limited availability of autologous graft tissues, there are not enough materials for continuous reparation. Therefore, people are more and more interested in using new tissue engineering therapies to construct tympanic scaffolds with functions and 3D geometry. At present, a primary tympanic membrane with a three‐layer composite scaffold has been constructed, which has both mechanical and conductive properties of the tympanic membrane.^[^ ^10^ ^]^ However, it is still a challenge for patients to fully recover their hearing ability because of the accurate arrangement of collagen fibrils in the primary tympanic membrane and the loss of related vibration behavior.

#### 2.3.2 Stapes Surgery

Stapes is a small stirrup‐shaped bone attached to the oval window membrane, which is responsible for transmitting vibration to the oval window membrane of the inner ear. Otosclerosis, tympanosclerosis, or congenital malformation can lead to the fixation of stapes, making it lose its function of transmitting sound. The fixation of the stapes footplate will lead to CHL and even sensory nerve loss caused by the infection of the inner layer of the eardrum.^[^ ^81^ ^]^ Stapedial surgery can be performed by stapes footplate resection, stapes footplate fenestration, or total stapedial resection and prosthesis implantation to treat hearing loss. With the introduction of laser‐assisted photoporation technology, the safety of stapes surgery has been improved. For example, the stapedial tendon and stapedial posterior arch were severed by hand‐held CO_2_ laser, and the stapedial footplate was fenestrated. Compared with the traditional manual drilling and windowing with a three‐edged needle, the method has the advantages of accurate positioning, less trauma, and fewer postoperative complications.^[^ ^82^ ^]^

#### 2.3.3 Fenestration of Inner Ear

Fenestration of the inner ear was first used to treat otosclerosis. Fenestration of the inner ear is to open a bone window on the external semicircular canal, so that sound can be transmitted to the inner ear through the bone window. It can be provided for patients with severe lesions that are not suitable for stapes surgery and some congenital malformed oval window membrane defects. At present, based on the surgery, the release of drugs in the inner ear can be realized. By selecting the osmotic pumps with different flow rates and different durations, the drugs can be accurately and controllably released in the inner ear of patients, while reducing the side effects of systemic medication. Combined with microporous technology, the osmotic pump can control the aperture of the window in a small range, and the drugs can be stably and accurately released.^[^ ^83^ ^]^

**Figure 5:**
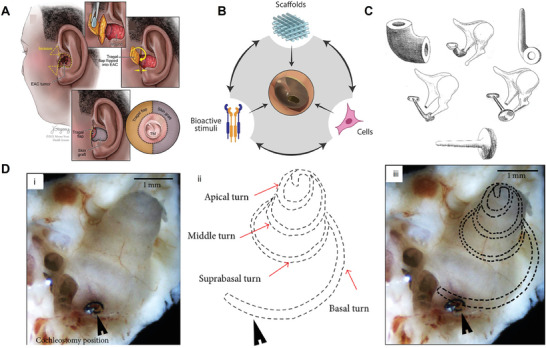
Hearing reconstruction operation chart. A) Air grid flap for surgical reconstruction of the external auditory canal.^[^ ^85^ ^]^ Reproduced with permission. Copyright 2021, Elsevier Inc. B) Regenerative therapies for the TM. Schematic diagram of three TE pillars applied to a perforated eardrum.^[^ ^10^ ^]^ Reproduced with permission. Copyright 2022, Elsevier Ltd. C) Various designs of reconstruction prosthesis with ceramic ossicular chain in vitro collected by the author are part of the extensive research results of ceramic implants in vitro. i, ii) Incudostapediopexy bio‐vitroceramic prosthesis. iii, iv) maleovestibulopexy bio‐vitroceramic prosthesis; v, vi) miringovestibulopexy bio‐vitroceramic prosthesis.^[^ ^86^ ^]^ Reproduced with permission. Copyright CC‐BY‐4.0. D) “Analysis of the Damage Mechanism Related to CO_2_ Laser Cochleostomy on Guinea Pig Cochlea” Cochleostomy was performed in the basal turn near the round window. i) The perforation as the black arrow showed the cochleostomy position. ii) The cochlear membrane was delineated with dotted lines. The red arrows located the specimens from the basal turn, suprabasal turn, middle turn, and apical turn. iii) The site of the simulated cochlear membrane under the bone. Scale bar: 1 mm.^[^ ^87^ ^]^ Reproduced with permission. Copyright CC‐BY‐4.0.

To ensure the hearing reconstruction is successful, the doctors need to choose the most suitable personalized surgical method and transplant materials through the comprehensive analysis of the patient's general condition, audiological examination results, and lesion scope. Meanwhile, because the patient's condition is ever‐changing, doctors need to adjust the plan at any time during the operation to implement personalized and precise treatment, so as to maintain or improve the patient's hearing to the greatest extent and reduce the probability of hearing loss. Otolaryngology surgery is delicate, with complex anatomical structures, important nerves, and blood vessels (**Figure** **5**). In order to reduce the postoperative complications of patients with hearing reconstruction, doctors need to know the fine anatomical structure of the middle ear before the operation, to facilitate the design of surgical schemes and the evaluation of the postoperative effect. The surgery often needs high‐resolution CT scanning technology and facial nerve monitoring technology. Further improved microsurgical skills, can make hearing reconstruction relatively simple, safe and effective, and reduce postoperative complications.^[^ ^84^ ^]^

### 2.4 Cochlear Implants

Although hearing aids can help patients with mild to moderate hearing loss, the information transmission between hair cells and the auditory nerve is interrupted and the functions of hearing aids are limited when the patients' hair cells are completely lost. Therefore, it is necessary to explore new methods and devices. CI is a device that can bypass the damaged hair cells and directly stimulate the auditory nerve to restore hearing. The first successful implantation of CI can be traced back to 1957. Djourno and Eyries in France implanted electrodes in the inner ear of a deaf patient for the first time, which gave him a sense of hearing.^[^ ^12^ ^]^ In 1972, the US House‐3M single‐channel CIs became the first generation of commercial devices. In 1982, the Australian Nucleus 22 CI was approved by the FDA and became the first multi‐channel cochlear device in the world. In the early days, the influence of CIs technology on residual hearing had not been studied, CI was only used for patients with sensorineural deafness who had completely lost their hearing function. Until 1995, CI was approved for adults with severe deafness and patients with residual hearing.^[^ ^88^ ^]^ The effect of CIs on patients was recognized by the National Institutes of Health (NIH) and the CIs became more common in adults and children with residual hearing. The CIs have changed the treatment of SNHL and introduced strategies other than amplification to improve the success rate of hearing recovery.

The CI system consists of the external audio processor and the implants that bypass the auditory nerve. The CI picks up the sound through the microphone and transmits it to the audio processor. Then, the audio processor amplifies, filters, and digitizes the sounds of the microphone. Finally, the meaningful information was filtered out. These messages are encoded and transmitted into the body by transmitting or socket transmission, and then received by the signal receiver in the body and converted into electric pulses, which directly stimulate SGNs to produce hearing. Electrical pulses are generated by 12–22 electrode arrays embedded in the conductive lymphatic vessels of the cochlea.^[^ ^89^ ^]^ The CIs can simulate the propagation process of high and low‐frequency sounds in normal hearing. High‐frequency sound propagates through the displacement of the cochlear basement membrane and fluid, while low‐frequency sound produces stronger displacement to the top of the cochlea. Each electrode of CIs will stimulate the frequency‐related part of the auditory nerve. The long electrode array can be inserted deeper to achieve low‐frequency stimulation, and the short array can be inserted into the base of the cochlea to protect the high‐frequency residual hearing at the tip of the cochlea.

However, the gap between the electrode and the auditory nerve, and the different causes the users make the CIs users cannot get the best listening experience. For example, the speech recognition ability is poor under background noise, and the CIs users cannot obtain satisfactory music perception. The possible reasons include the small number of electrodes, extensive neuronal activation caused by the diffusion of electrode current in the cochlea, unexpected interaction between channels, and the limitation of CIs in the fine time structure of transmitting acoustic signals.

**Figure 6:**
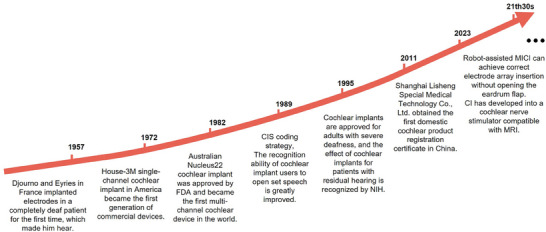
Development history of CIs.

Since 1993, Lehnhardt put forward the soft surgery technique, which can make patients retain a certain degree of residual hearing during implantation^[^ ^90^ ^]^ (**Figure** **6**). With the development of research on residual hearing preservation, the concept of minimally invasive cochlear implantation (MICI) has gradually formed. At present, the research showed that MICI assisted by the robot can realize correct electrode array insertion without opening tympanic membrane flap.^[^ ^91^ ^]^ Simultaneously, some CIS users have indicated that their external receivers are easy to fall off and it is inconvenient to do strenuous exercise after wearing them. Jang et al. designed a triboelectric‐based artificial basilar membrane (TEABM) to simulate the tension displacement of the cochlea, which has the advantage of a self‐powered energy supply without any external batter.^[^ ^92^ ^]^ This study also envisaged the development of a self‐powered and miniaturized TEABM as a substitute for traditional CI, so as to realize the miniaturization of CIS and solve the problem that external receivers are easy to fall off. Some newer technologies even allow the CIS to measure the potential during insertion.^[^ ^93^ ^]^ Magnetic resonance imaging is widely used in the diagnosis of clinical diseases because of its high resolution on soft tissues, safety, and no radiation.^[^ ^13^ ^]^ However, the early single‐channel cochlear nerve stimulator is incompatible with MRI because of the existence of magnets. Now it has developed into a cochlear nerve stimulator compatible with MRI. For example, the magnet in MED‐EL CIS is a rotating magnet, which can rotate and align freely according to the magnetic field scanned by MRI. Therefore, it is safe and feasible for patients with this type of cochlear implant to use a 1.5T magnetic resonance imaging device to monitor the internal auditory canal after implantation.^[^ ^94^ ^]^

The CIs in the future may represent the transformation of nerve stimulation mode. At present, a lot of research is exploring more complex cochlear nerve stimulation technology, including piezoelectric stimulators without external power supply,^[^ ^95^ ^]^ and optical CIs using optogenetics nerve stimulation mechanisms.^[^ ^96^ ^]^ The goal of these new technologies is to improve the signal integrity transmitted to the cochlear nerve. With the further improvement of the series of works, it has great potential in the treatment of patients with hearing loss in CIS.

## 3 Mechanism of Physical Stimulation in Promoting SGNs Regeneration

Except for a few cases of sensorineural deafness are based on auditory pathways or cortical lesions, most of them are caused by the loss or functional defect of hair cells. Hearing aids are effective for patients with mild to moderate and partial severe deafness, but they are not effective for most patients with severe, extremely severe, and even total deafness. Although patients often retain some spiral nerve cells and auditory nerve fibers, the number of functional SGN is insufficient. Though the effect of CIS is better than hearing aids, the effect of CIS depends entirely on the remaining functional SGN. Therefore, the repair and regeneration of SGNs is very important. Many studies have shown the importance of physical stimulation to the growth and development of neurons. The related researches are summarized from three aspects: the influence of electrical stimulation on SGNs, the influence of magnetic stimulation on SGNs, and the influence of light stimulation on SGNs.

### 3.1 Mechanism of Electrical Stimulation in Promoting SGNs Regeneration

**Figure 7:**
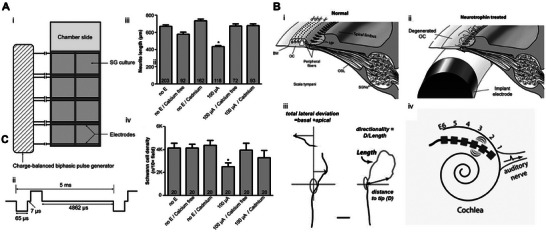
Mechanism of regeneration of SGN by electrical stimulation. A) Schematics of spiral ganglia (SG) dissociated explant culture with charge‐balanced biphasic electrical stimulation.^[^ ^98^ ^]^ i) Eight‐well chamber slides were used in SG isolation culture or cochlear transplantation culture. Four holes are opened on two opposite walls of each chamber near the floor to introduce two platinum‐iridium wires at two opposite boundaries. The wire is connected to the charge balance biphase pulse generator. ii) Biphasic pulse used for electrical stimulation has an amplitude of 50 A or 100 A, a pulse width of 65 s, an open‐circuit interphase gap of 8 s, a short‐circuit phase of 4862 s, and a frequency of 200 Hz. The decrease of calcium influx in cadmium or calcium‐free medium weakened the inhibition of neurite extension and the decrease of Schwann cell density. iii) In the culture stimulated by 48 h/100 A, the length of neurites treated with cadmium or calcium‐free medium is longer than that without these treatments. iv) The density of Schwann cells treated with cadmium‐free or calcium‐free medium is higher than that without these treatments.*p < 0.05 compared with all other groups in the same figure, Kruskal‐Wallis one‐way ANOVA on ranks followed by Dunn's method. Reproduced with permission. Copyright 2016, Elsevier Ireland Ltd. B) i)Schematic diagram of the sensory epithelium and SGNs in normal guinea pig cochlea showing the highly radial projection of the peripheral neurites from their SGN soma to the sensory hair cells located in the organ of Corti (OC).^[^ ^101^ ^]^ The peripheral neurites pass through the habenula perforata (HP), which is a small opening in the osseous spiral plate (OSL), where most neurites form synapses with the nearest inner hair cell (IHC). For clarity, the tectorial membrane and Reissner's membrane are not shown in this diagram. ii) Schematic diagram of a deafened cochlea showing a part of the electrode array in the scala tympani (ST) and loss of hair cells and OC after deafness. Normally, cochleae like this will show a significant reduction in the number of peripheral fibers and the loss of SGNs. iii) Example trajectories showing the derivation of path quantification measurements of lateral deviation, total neurite length, and directionality. iv) Schematic diagram of the six‐ring stimulation electrode array implanted in the deaf cochlea. Stimulating specific electrode pairs excite different subpopulations of SGNs across the cochleotopic gradient (e.g., E2–3). This cochleotopic output of SGNs forms the basis of cochleotopic sexual excitation mode in the auditory pathway, including in the ICC. Reproduced with permission. Copyright 2013, Wiley Periodicals, Inc.

For patients with severe hearing loss, the loss of hair cells is an important cause of their illness. CI is a good choice for treatment, which can bypass the damaged hair cells and regulate SGNs behavior by electrically stimulating SGNs,^[^ ^97^ ^]^ CI is the generator of electrical stimulation, but there is still controversy about the influence of electrical stimulation on nerve behavior. As shown in **Figure** **7**A, the research of Shen et al. proved that the neurite length of SGNs was shortened at 100 µA electrical stimulation intensity after 8 h of charge balance biphasic electrical stimulation in vitro, and the neurite extension was significantly inhibited by 50 µA electrical stimulation for 24 h.^[^ ^98^ ^]^ It indicated that the electrical stimulation was to inhibit the nerve extension of SGNs through calcium influx of various types of voltage‐dependent calcium channels (VDCCs). Kopelovich et al. indicated that the delayed hearing loss of CI implanters might be due to the excitotoxicity of electrical stimulation.^[^ ^99^ ^]^ Li et al. proved that although electrical stimulation is safe and effective, it will cause changes in synaptic structure.^[^ ^100^ ^]^ High‐intensity and long‐term electrical stimulation of 2 kHz could reduce the excitability of the auditory nerve, and high‐intensity electrical stimulation could reduce the number of synapses between inner hair cells and afferent SGNs. The research of Landry in Figure 7B showed that electrical stimulation can increase the neurite length of SGNs in animals.^[^ ^101^ ^]^ At present, in order to explore the regulation of electrical stimulation on the growth of SGNs, a lot of research systems of electrical stimulation in vitro have been developed. Peter et al. developed a special device for in vitro stimulation.^[^ ^102^ ^]^ It can select the parameters of different charge densities for a single cell in one experiment and can be used to study the damage of electrical stimulation to SGNs in vitro. The experiment showed that the change in the short pulse width of the current has a slight positive trend on the survival of SGNs and the growth of neurites. Liao et al. developed an efficient electroacoustic stimulation (EAS) system for CIs, in which the electrical stimulation system combined CI and conductive Ti_3_C_2_T_X_ Mxene—matrigel hydrogel.^[^ ^39^ ^]^ The results of electrical stimulation showed that low‐frequency electrical stimulation could promote the development of SGNs growth cones and neurites, as well as the transmission of intercellular signals. It could accelerate the signal transmission of SGNs induced by Ca^2+^ in vitro. In summary, electrical stimulation plays an important role in the study of SGNs, and most of the related applications of electrical stimulation research focus on CIs. However, the fundamental purpose is to regenerate or restore the function of SGNs, so as to restore the hearing of patients.

Studies showed that neural stem cells will migrate to the cathode at a higher rate in physiological intensity under the stimulation of an electric field.^[^ ^103^ ^]^ The mechanism of electrical stimulation applied on neural stem cells includes regulating the secretion of growth factors (NGF, BDNF, VEGF, neurite growth inhibitors, and nerve cell adhesion molecules), through the cell cycle by increasing the number of cells entering the S phase, increasing the level of intracellular calcium and the activation of proliferation signal pathways (such as ERK1/2 pathway and PI3‐Akt pathway).^[^ ^40^ ^]^ Some reports showed that low‐frequency sinusoidal electrical stimulation can regulate membrane matrix, cytoskeleton reorganization, and cell surface receptor redistribution.^[^ ^64^ ^]^ This may regulate the differentiation of neural stem cells and the growth of neurites by changing the protein conformation of the extracellular matrix (ECM) or intracellular signaling pathway.^[^ ^104^, ^105^ ^]^

### 3.2 Mechanism of Photostimulated Regeneration of SGN

#### 3.2.1 Infrared Nerve Stimulation(INS)

During the decades of INS’ development, researchers have explained the complex mechanism of INS applied to hearing restoration from different perspectives. In the discussion about the main mechanisms of INS in the treatment of hearing loss, the academic community generally agrees on the following two effects. The first one is the photo‐thermal effect. These include the electrostatic mechanism by which INS reversibly alters the capacitance of the cytoplasmic membrane to depolarize the target cells,^[^ ^44^, ^106^, ^107^, ^108^ ^]^ and the IR laser‐evoked response mechanism in sensory neurons mediated by the TRPV4 thermal channel.^[^ ^109^, ^110^ ^]^ The other is the photomechanical effect. SGNs can transmit action potentials through stress relaxation wave stimulation of hair cells with the photoacoustic mechanism.^[^ ^111^, ^112^, ^113^, ^114^ ^]^ Nevertheless, there is still a need to confirm the primary mechanism through additional studies to clarify the applicable hearing loss pathology (INS acting directly on auditory neurons or residual functional hair cell‐mediated post‐deafness action on auditory neurons) for INS in clinical trials.

Wells et al. systematically studied the biophysical mechanisms of transient photostimulation of peripheral nerves in 2007^107^. The rat sciatic nerve was stimulated with a holmium yttrium aluminum garnet laser (2.12 µm), a free‐electron laser (2.1 µm), an alexandrite laser (750 nm), and a prototypical solid‐state laser neurostimulator (1.87 µm) in order to confirm the contribution of photo‐thermal, photo‐mechanical, photo‐electric‐field, and photo‐chemical effects in photostimulation. The results showed that the most important reason for realizing transient photostimulation of peripheral nerves was the photo‐thermal effect. It means the direct or indirect activation of cellular transmembrane ion channels induced by thermal transients, which results in the generation of action potentials. The secondary cause may be a photomechanical effect. Shapiro et al. have further confirmed the specific mechanism of the photo‐thermal effect.^[^ ^106^ ^]^ The infrared laser pulse absorbed by water produced a rapid and localized temperature increment, which could transiently increase cell membrane capacitance and cause the cell to generate a depolarizing current. Some changes in the corresponding electrophysiological response to IR stimulation in the model of African Xenopus oocytes, cultured mammalian cells were also observed by the team. Moreover, in a model of an artificial lipid bilayer with extremely simplified functionality, the same idea about the mechanism of IR stimulation was further verified. Thereafter, in the study of Izzo, a diode laser with the *λ* of 94 µm and stimulation pulses as low as 5 µs was used to stimulate the auditory system of gerbils.^[^ ^44^ ^]^ By choosing a different wavelength of stimulation than previous studies, the team concluded that the primary role in controlling auditory nerve stimulation was a water‐mediated photo‐thermal effect in the tissue.

However, as the study progressed, many researchers discovered experimental phenomena that underpinned the photo‐mechanical effect as the primary mechanism. Unlike other sensory neurons, auditory neurons are located in the cochlea and can receive electrophysiologic signals encoded from inner hair cells. The hair cells have extremely sensitive mechanosensitive channels, which makes the photo‐mechanical effect a non‐negligible role in auditory nerve stimulation. Teudt et al. proposed that in a thermally constrained state, even if the energy used to stimulate neurons is low, rapid heating of an optical fiber immersed in water can cause that volume to expand and generate stress relaxation waves.^[^ ^114^ ^]^ In turn, these stress relaxation waves may stimulate residual functional hair cells in animal models of hearing loss, producing stimulation of the auditory nerve. Thompson et al. challenged previous work suggesting that the photo‐thermal effect is the primary mechanism of INS.^[^ ^113^ ^]^ They argued that most of the previous studies were carried out based on animal models with either normal hearing or incomplete hearing loss, which could not ensure that the INS stimulates the auditory nerve without the involvement of hair cells. Even when different deafening techniques were adopted, there were still differences in the constructed hearing deficits, and there was no guarantee that the neural activity induced by INS was a direct interaction between the laser and the SGN. They altered the deafening procedure in their experiments to cause severe profound deafness in the animal model. Histological analysis of the cochlea was performed after this procedure to ensure the loss of functional hair cells in the model. The results showed that electrically evoked Auditory Brainstem Response (eABR) could be easily evoked by electrical stimulation in all cochleae after deafness, but optically evoking an auditory brainstem response (oABR) was weak. The experimental results provided support for the theory that stimulation of the INS in the cochlea may be mediated by hair cells. In conclusion, the discussion on the primary mechanisms still continues and a great deal of research needs to be invested in the future to clarify the use of INS in the treatment of hearing loss pathologies.

#### 3.2.2 Optogenetic

**Figure 8:**
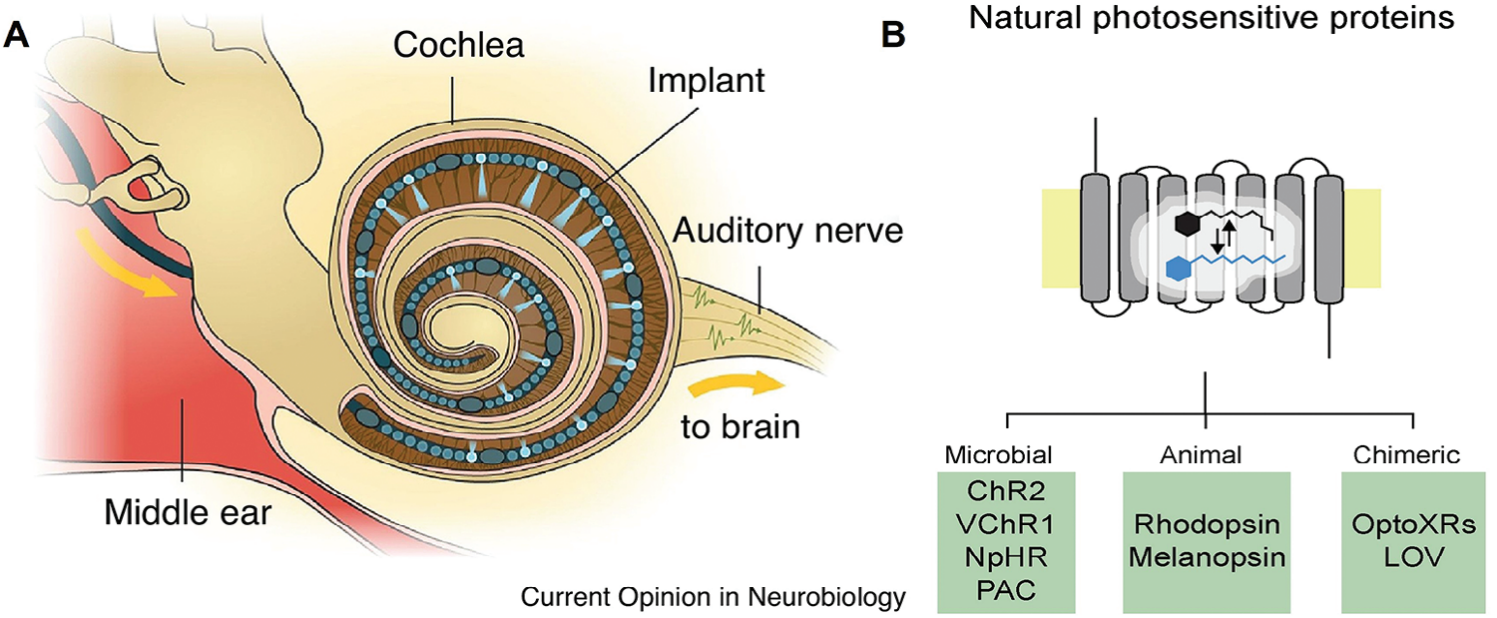
Related studies of optogenetic mechanisms. A) Optical SGN stimulation, such as lenses focusing light emitted by micro light‐emitting diodes (µLEDs) or light emitted by waveguide arrays, is expected to spatially limit the activation of SGNs, thus allowing for more independent stimulation of the channels and improving the frequency and intensity resolution of the encoding of sound.^[^ ^121^ ^]^ Reproduced with permission. Copyright 2015, Elsevier. B) Photochemical tools for auditory neuron manipulation – photosensitive proteins.^[^ ^114^ ^]^ Reproduced with permission. Copyright 2009, Elsevier.

Optogenetics is an emerging multifunctional tool for cellular‐level manipulation. As shown in **Figure** **8**, the basic principle is that the photostimulation of a class of photosensitive ion channels in the cell membrane can achieve modulation of the cell membrane potential, thereby eliciting action potentials in the target cell.^[^ ^115^ ^]^ This class of membrane proteins based on the activation of photosensitive ion channels is known as type I retinoids. One of the most commonly used optic proteins is channel retinal (ChR). The discovery of two types of channel retinoid isoforms in the green alga Chlamydomonas reinhardtii opened the door to optogenetics.^[^ ^116^ ^]^ In particular, the first successful transfection of mammalian neurons with ChR2 broadened the scope of optogenetics in neuromodulation and made it possible to apply optogenetics to humans.^[^ ^117^ ^]^ Optogenetics has the advantages of reliability, rapidity, temporal precision, and noninvasiveness. It has made the technique show good potential for development in DBS,^[^ ^118^ ^]^ fluorescence imaging,^[^ ^119^ ^]^ vision restoration,^[^ ^120^ ^]^ and other research areas. In the field of hearing restoration, CIs based on optogenetic stimulation have emerged as the most promising alternative to conventional electrical CIs. Research related to optical cochlear implants (oCIs) has also become the frontier of hearing restoration research.^[^ ^121^ ^]^

### 3.3 Mechanisms of SGN Regeneration By Magnetic Stimulation

Among the various physical stimuli acting on SGN, magnetic stimulation has very prominent characteristics. Whether it is direct‐current (DC) MF or altering‐current (AC) MF, it has strong penetration to biological tissues, which means the effect of MF will not be attenuated by the depth changes of the target point. At the same time, with a strategy based on functionalized MNPs, it is able to achieve remote intervention as well as non‐invasive treatment for SGNs. Therefore, all these features are extremely attractive in the treatment of inner ear diseases.

#### 3.3.1 Direct Uptake of SPION

Neural regeneration is a major challenge in neuroscience for treating degenerative diseases and repairing damaged nerves. Studies have shown the importance of physical stimulation for neuronal growth and development. Responding to magnetic stimulation via well‐established MNPs is currently the most common approach for nerve regeneration. Superparamagnetic iron oxide nanoparticles (SPION) are considered to be one of the most promising materials. Superparamagnetic nanoparticles are nanoscale particles that are magnetically responsive and generally have a diameter of <30 nm.^[^ ^123^ ^]^ They generally consist of an internal magnetic core and an external polymer coating. The internal core is made of iron oxide and is used to respond to the external MF. The surface of SPION is usually coated with a strongly stable polymer coating, including materials such as polyvinyl alcohol, polyethylene glycol, dextran, starch, and poly (L‐lysine) (PLL). These polymer coatings are used to enhance the biocompatibility of SPION and to prevent polymerization, biodegradation, and structural changes when SPION is exposed to specific environments. SPION is modified to bind to the receptors/ligands of different drugs or target cells through electrostatic interactions on the surface or covalent couplings. It aims to achieve improved drug effects and enhanced targeting. The magnetization strength of SPION is highly correlated with the external MF. This property makes it possible to guide and manipulate the nanoparticles by applying an external MF, which in turn affects the interaction of the cells with the MNPs. Another outstanding advantage of SPION is that it provides a method for high‐precision, non‐invasive tracing of target cells. This approach can provide important information about the effectiveness of cell therapy as well as the safety of the treatment. Based on the above advantages, SPION has important research value in the fields of neural repair therapy,^[^ ^124^, ^125^ ^]^ drug delivery,^[^ ^61^, ^126^ ^]^ and cell labeling tracing.^[^ ^127^ ^]^

**Figure 9:**
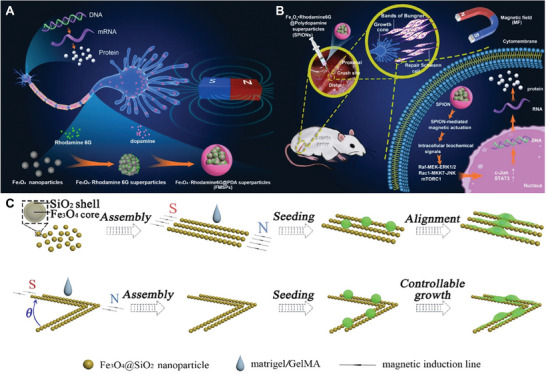
Magnetic stimulation of nerve regeneration mechanism studies. A) Fe_3_O_4_‐Rhodamine 6G@polydopamine superparticles (FMSPs) as a novel stimulator to promote non‐invasive nerve regeneration via cellular magnetic actuation.^[^ ^128^ ^]^ Reproduced with permission. Copyright 2020, Springer Nature. B) Schematic representation of SPION‐mediated magnetic activation to promote neural regeneration by inducing and maintaining a repair‐supportive phenotype in ceviche cells.^[^ ^129^ ^]^ Reproduced with permission. Copyright 2022, Springer Nature. C) Schematic representation of a scheme in which magneto‐colloidal nano‐chains guide the growth of SGNs^49^. Reproduced with permission. Copyright 2022, Elsevier.

As shown in **Figure** **9**A,B, Neuronal cells can directly ingest SPION to exert the corresponding biological effects.^[^ ^128^, ^129^ ^]^ It has been found that the NGF family can enhance nerve regeneration when shared with SPION, and the mechanism by which SPION stimulates neuronal growth and development is complex. It has been hypothesized that it is related to its intracellular production of iron ions after endocytosis by cells.^[^ ^130^ ^]^ Due to endocytosis, the nanoparticles entered into lysosomal vesicles. Low pH in the vesicles can promote the release of Fe ions. The increase in intracellular iron ions affected the activity of extracellular signal‐regulated kinase (ERK), which led to an increase in ERK activity, thus promoting neurite growth in PC12 cells. Another study showed that SPION activated the mitogen‐activated protein kinase (MAPK) signaling pathway, which stimulated the neural differentiation of PC12 cells.^[^ ^131^ ^]^ Fe, as a strong oxidizing agent, can stimulate reactive hydroxyl free radicals. Some researchers have hypothesized that it has the same effect as Mn as a neuroenhancer and could stimulate nerve growth by inducing the generation of reactive oxygen species.^[^ ^132^, ^133^, ^134^ ^]^

Meanwhile, cellular tension has also been suggested to be an important factor in the influence of SPION on neuronal growth and orientation.^[^ ^48^, ^135^ ^]^ The combination of SPION and external MF can not only promote neurite growth but also guide neurites to grow preferentially parallel to the MF direction. The ability of cell migration is highly correlated with nerve regeneration, and the effect of SPION on cell migration is highly controversial. According to the results of Soenen,^[^ ^136^ ^]^ when SPION is internalized by neurons, the neuronal cell actin cytoskeleton and microtubule network are disrupted, leading to the loss of adhesive patches and a decrease in cell migration ability. In contrast, another study showed that the internalization of SPION had no significant effect on the migration of ceovan cells compared with the control group.^[^ ^137^ ^]^ In a study by Hu^[^ ^48^ ^]^ and his cooperators, SPION significantly inhibited the migration of SGNs compared to untreated cells. However, SGNs internalizing SPION exhibited enhanced migration when external MF was applied compared to the SPION group. Thus, the SPION‐induced reduction in migration ability could be reversed by applying external MF.

#### 3.3.2 Creation of a Magnetically Responsive Bionic Extracellular Matrix

Extracellular Matrix (ECM) refers to the non‐cellular portion of a tissue that has a 3D mesh structure composed of bioactive macromolecules. In recent years, with more in‐depth studies, researchers have found that the extracellular matrix of the nervous system plays an important role in neural development and regeneration processes. The ECM mediates neural development and regeneration processes through the interactions of cells and ECM, including the maintenance of the ecological niche of stem cells, neuronal migration, and neuronal synaptic growth.^[^ ^138^ ^]^

The ECM is composed of five main types of substances, namely collagen, non‐collagen, elastin, proteoglycans, and aminoglycans. The presence of these biologically active macromolecules gives the ECM a biological function of interacting with cells while also conferring physical properties such as morphology and stiffness. These physical property cues play equally important roles in neurogenesis, neural repairing, and neuronal cell migration.^[^ ^139^ ^]^ Among these different biophysical properties, matrix elasticity and morphology have been well studied and proved to greatly influence neuronal cell behavior (Figure 9C).^[^ ^49^, ^126^ ^]^

The viscoelasticity and topography of the ECM can influence many different behaviors of neuronal cells, including neuronal migration, neuronal stem cell fate regulation, and neural regeneration. Therefore, constructing biomimetic ECM environments in vitro plays a crucial role in studying neuronal cell behavior. Currently, biomaterials such as hydrogels, graphene oxide, and electrostatic spinning have been continuously optimized and applied to develop biomimetic platforms. The addition of magnetic nanomaterials to these biomaterials is one of the high‐profile optimization methods. Magnetic nanomaterials have nanoscale pore sizes and can be uniformly dispersed in biomaterials such as hydrogels through cross–linking or in situ formation. By doping the magnetic nanomaterials, the biomaterials become magnetically responsive. By applying an external MF, the shape and stiffness of the biomimetic platform can be changed to affect the occurrence and variation of nerve cell behavior. Furthermore, magnetic stimulation can be combined with wireless stimulation, such as electrical stimulation and optical stimulation, to provide a more complex multilayer stimulation bionic platform for nerve cell regeneration.

#### 3.3.3 Neuromodulation

Neuromodulation is a biomedical engineering technology that utilizes invasive or non‐invasive technology and adopts physical (light, magnetic, electric, ultrasound) or chemical means to change the functions of the nervous system. The nervous system acts as a “transmission network” for various signals in humans, and relies on action potentials for information transfer and regulation of homeostasis. The generated action potentials after the cells are effectively stimulated will cause the opening of corresponding gates in the cells, triggering the corresponding ion channels to generate ion flow, and thus enabling the cells to respond to various signals for the transmission of information. The gating stimuli can be diverse, such as thermal transient receptor potential (TRP) channels and pressure‐sensing piezoelectric channels. Many ion channels contain modular structural domains that can directly sense the stimulus and then modulate ion channel switching through structural domain‐structural domain interactions. These ion channel families are particularly sensitive to exogenous signals such as light irradiation, acoustic waves, electric currents, thermal effects, mechanical forces, and MFs. Therefore, neuromodulation can be achieved by applying external signals to target the corresponding ion channels. It is known that optogenetics can provide cell population‐specific neuromodulation with high temporal precision. Accordingly, the concept of magnetogenetics exists in the field of magnetic stimulation. Researchers use techniques such as cell transfection to enable targeted cells to establish consistently uniform levels of ion channel expression. MF energy can easily penetrate tissues and be converted by nanoparticles (including piezoelectric, mechanical, temperature, etc.) into a bio‐stimulus that activates the corresponding ion channels. It provides the possibility of remote modulation of neurons by magnetic stimulation.

## 4 Application of Physical Stimulation in Promoting SGNs Regeneration

### 4.1 Applications of Electrical Stimulation in Promoting SGNs Regeneration

The existing research work on the electrical stimulation of SGNs were summarized into three parts: the regulation of SGNs, the promotion of stem cells to regenerate SGNs, and the protective effect on SGNs

#### 4.1.1 The Regulation of SGNs

Electrical stimulation is a common strategy to regulate cell behavior in vivo and in vitro. Electricity is one of the important factors in regulating signal pathways and inducing cell behavior. Inducible cell behaviors include proliferation and migration in pathophysiological processes, such as wound healing, embryonic development, and tissue regeneration.^[^ ^140^ ^]^ At present, the research on cell regulation by electrical stimulation mainly focuses on stimulating receptors and ion channels on cell membranes to drive proliferation, hyperpolarization, depolarization, differentiation, and migration. Electrical stimulation can accelerate nerve regeneration and target nerve regeneration after nerve injury by promoting axon growth of damaged neurons. The study showed that electrical stimulation at the proximal end of the injury site for 1 h and 20 Hz can promote the expression of endogenous growth factors, thus accelerating axon growth and target nerve regeneration.^[^ ^141^ ^]^ At the same time, a study proved that short‐term electrical stimulation can effectively accelerate the growth of axons at the nerve injury site, and the short‐term electrical stimulation of axons near the nerve injury site is essential for the acceleration of axon growth in response to electrical stimulation.^[^ ^142^ ^]^ Because the effect of electrical stimulation on nerve excitability depends on the frequency of stimulation. When the frequency is greater than 1khz, it will lead to nerve habituation and reduce the excitability of neurons at this stimulation intensity.^[^ ^143^ ^]^ Li's study showed that kilohertz frequency alternating current can inhibit neurons at kilohertz frequency. Depolarization and sodium channel inactivation can induce electrical nerve block. The inhibition of nerve conduction will last for 4 h after 1 h of electrical stimulation which is higher than the threshold of electrically induced compound action potential of 3db.^[^ ^99^ ^]^

**Figure 10:**
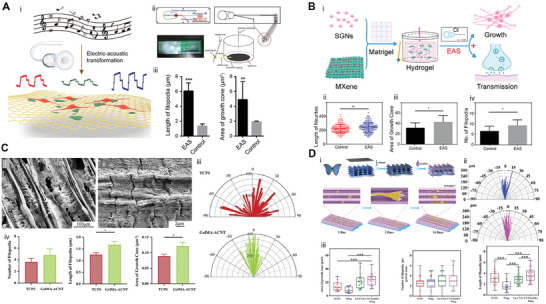
Electrical stimulation regulates SGNs. A) i) Electro‐acoustic stimulation of SGNs based on cochlear implant.^[^ ^144^ ^]^ ii) Construction of the cochlear implant/graphene EAS system. iii) Histogram of filament length and growth cone area of SGNs cultured with or without EAS. Reproduced with permission. Copyright 2019, American Chemical Society. B) i) 3D Ti3C2Tx MXene−Matrigel with Electroacoustic Stimulation to Promote the Growth of SGNs^39^. ii) Average neurite length of SGN in the control group and EAS group. The unit of the ordinate is micrometers (µm). iii) Statistical graph of the number of filopodia. iv) Statistical graph of the filopodia length. The unit of the ordinate is micrometers (µm). Reproduced with permission. Copyright CC BY‐NC‐ND 4.0. C) The low‐magnification i) and high‐magnification ii) SEM image of the GelMA‐SACNT substrate.^[^ ^156^ ^]^ iii) Representative polar histograms of neurite growth on the TCPS and GelMA‐SACNT. iv) The number and length of filaments and the area of growth cones of SGNs cultured on the TCPS and GelMA‐SACNT substrates. *p < 0.05. Reproduced with permission. Copyright 2022, Elsevier. D) i) Topographically Conductive Butterfly Wing Substrates for Directed SGN Growth.^[^ ^161^ ^]^ ii) Orientation angle distribution of SGNs on M. Menelaus wings and CNT/GelMA‐integrated wings. iii) Average growth cone area. One‐way ANOVA, ***p < 0.001. Average number of filopodia per growth cone. One‐way ANOVA. Average filopodia length. Reproduced with permission. Copyright 2021, Wiley‐VCH.

Simple electrical stimulation has a poor ability to regenerate SGNs, but its combination with conductive materials or other stimuli can better regulate the regeneration of SGNs.^[^ ^40^ ^]^ Conductive graphene films are not only highly biocompatible but also can regulate the bioelectricity of cell membranes by enhancing the micro‐electric field generated by cells themselves. So, graphene has been widely used in the research of tissue engineering (**Figure** **10**A).^[^ ^144^ ^]^ Guo et al. used graphene film as the conductive substrate of the EAS system and explored the influence of electrical stimulation of CI on SGNs.^[^ ^40^ ^]^ The results showed that long‐term and discontinuous EAS may promote the development of SGNs neurites and significantly accelerate the development of growth cones and filamentous feet. Although the research proved that the system can be used as an effective method to adjust SGNs, it is necessary to design a 3D conductive biomaterial as a cell carrier, and the functions of regenerated neurites in vivo need further verification.

MXene and its derivatives have also been widely used to construct the microenvironment of nerve cells. The general formula of MXene is Mn+1XnTx (the range of *n* is 1–4), in which M is a transition metal, X is carbon or nitrogen, and Tx is a rich functional group at the surface end of the outer transition metal layer.^[^ ^145^ ^]^ Its thickness is only a single atom or a few atoms. Its transverse dimension depends on the preparation method of the material, which can reach several microns or even more. MXene can be synthesized by a top‐down or bottom‐up strategy. The typical top‐down method is to selectively peel off the element A layer from the precursor and transform it into an MAX phase or a non‐MAX phase. The bottom‐up method is not suitable for biomedical engineering because of its shortcomings such as low yield and lack of surface modification ability.^[^ ^146^ ^]^ Due to the large surface area, adjustable size, high conductivity, hydrophilic surface, and excellent mechanical properties, MXene and its derivatives have attracted much attention in biomedicine, such as regenerative medicine,^[^ ^147^, ^148^ ^]^ biosensor,^[^ ^149^, ^150^, ^151^ ^]^ drug delivery.^[^ ^152^, ^153^ ^]^ It has been found that Ti_3_C_2_T_x_ MXene can be used as a neural interface or scaffold material to promote the differentiation of neural stem cells (NSCs) and the growth of neurites.^[^ ^154^ ^]^ The material could selectively increase Ca^2+^ voltage‐gated current in neurons and enhance synaptic transmission between neurons. It provided a new direction for nerve interface or scaffold in nerve tissue engineering. As shown in Figure 10B, Liao et al. proposed an electrical stimulation system constructed by CIs and conductive Ti_3_C_2_T_x_ MXene‐matrigel hydrogel.^[^ ^39^ ^]^ The researchers cultured SGNs in Ti_3_C_2_T_x_ MXene‐matrigel hydrogel and exposed them to electric stimulation conducted by CIs. The result showed that the stimulation system could accelerate the signal transmission of SGNs induced by Ca^2+^ in vitro. SGNs signal transmission may enhance the signal through the connection between synapses. At the same time, the higher frequency of calcium oscillation events in this system further reflected the role of this system in promoting the formation of neural networks. After systematic stimulation, the expression of SGNs‐related genes such as ion transmembrane transport, synaptic transmission, synaptic plasticity, and cell adhesion were up‐regulated. It showed that low‐frequency stimulation could promote the growth cone development, neurite growth, and signal transmission between cells of SGNs. The system established in this work provided a new direction for the repair of SGNs and the improvement of the comprehensive technical system of CIs.

Because of the electrical characteristics of nerve cells and the positive regeneration response of nerve cells to electrical stimulation, many conductive materials have been used in the research of nerve tissue engineering. The gap between the electrode array of CIs and SGNs will affect the stimulation of neurons, and the directional growth of neurons can make up for this problem. Therefore, it is also very important to study the directional growth of neurons based on conductive materials. Super‐aligned carbon nanotubes are widely used in neurobiological research because of their excellent electrical conductivity, mechanical properties, and unique surface structure.^[^ ^155^ ^]^ Hu et al. assembled the super‐aligned carbon nano‐scaffold onto the biocompatible gelatin methacrylate hydrogel (Figure 10C).^[^ ^156^ ^]^ The composite material inherited the topological structure of super‐aligned carbon nano‐materials and the biocompatibility of hydrogels. The study showed that SGNs grown on composite materials have higher calcium activity, and the composite materials can promote the signal transmission of neurons. Simultaneously, the composite material could promote the growth and maturity of SGNs by adjusting the elongation of neurites with cell orientation and the development of growth cones. It had great potential in promoting the regeneration of SGNs. Morpho Menelaus butterfly wings can be used in electronic sensors,^[^ ^157^ ^]^ and photonic devices,^[^ ^158^ ^]^ it can also be used as a substrate to adjust cell orientation.^[^ ^159^, ^160^ ^]^ As shown in Figure 10D, Wei et al. assembled super‐aligned carbon nanotubes in parallel along the wings of the Morpho Menelaus butterfly, and covered them with biocompatible gelatin methacrylate hydrogel to construct composite materials.^[^ ^161^ ^]^ Because of its unique 3D topological structure, the super‐aligned carbon nanotube integrated wings could guide the growth direction of SGNs and promote the growth of spiral neuron neurites. At the same time, the excellent conductivity of this material provided a platform for the research of EAS stimulation systems based on SGNs. This study explored the potential of Morpho Menelaus butterfly wings as 3D layered composite conductive materials in cell repair, which provided a new direction for nerve repair.

To sum up, electrical stimulation combined with materials with special spatial structures can effectively regulate the related life activities of SGNs, and the clinical applications of these materials need further research.

#### 4.1.2 Promotion of Stem Cells to Regenerate SGNs

Transplantation of neural stem cells has become a new method to treat neurodegenerative diseases, including sensorineural deafness. In recent years, the replacement therapy of stem cells has made remarkable progress in the treatment of hearing loss.^[^ ^162^ ^]^ The stem cells are self‐renewal and have low immunogenicity, strong migration, and differentiation ability. It has the potential to restore hearing by implanting neural stem cells into the inner ear, regenerating SGN, and forming synaptic connections.^[^ ^163^ ^]^ The environment around stem cells determines their fate, such as biochemical factors, physical factors, and the interaction between cells. When conducting the research in vivo or in vitro, changes in the surrounding environment will have an impact on stem cells, and the correct regulation of stem cells will be more conducive to the development of stem cell therapy.^[^ ^164^ ^]^ Stem cells used for SGNs regeneration include Embryonic stem cells (ESCs), NSCs, Mesenchymal Stem Cells (MSCs), induced pluripotent stem cells, hematopoietic cells, and inner ear stem cells. However, due to the low survival rate and differentiation rate of many transplanted stem cells and the low efficiency of regenerating functional neurons, it is very important to clearly manipulate the behavior of stem cells.^[^ ^40^ ^]^ Under this premise, electrical stimulation is also effective in regulating stem cell behavior.

The regulation of electrical stimulation on stem cells depends on the microenvironment of stem cells to some extent. The traditional culture system is 2D, such as perforated plates, round coverslips, and Petri dishes. Although these basic 2D culture systems are valuable in basic research, they are not suitable for clinical research that requires a large number of cells for transplantation and regeneration.^[^ ^164^ ^]^ The 2D culture system makes it difficult to simulate the internal environment where cells live in vivo. Therefore, it is very important to establish a 3D culture system that can simulate the living environment of stem cells in vivo. Cheng et al. reviewed the effects of different conductive materials on the differentiation of neural stem cells.^[^ ^165^ ^]^ Conductive nanomaterials with complex 3D structures, such as conductive hydrogels, carbon nanotubes, and other nano‐materials were mentioned in the study. These 3D materials were more effective strategies for stem cell therapy. At the same time, because these materials can provide a suitable living environment for cells and are easily compatible with various stimuli, it is expected to be applied in vivo. At present, there are two main types of 3D scaffold materials: natural and artificial materials. Graphene materials are widely used in medical fields, such as tissue engineering,^[^ ^166^, ^167^, ^168^ ^]^ drug and gene delivery,^[^ ^166^, ^169^, ^170^ ^]^ biosensor,^[^ ^171^, ^172^ ^]^ and so on, because of their unique physical characteristics such as high charge mobility, good mechanical strength and good cell compatibility and biological safety.

**Figure 11:**
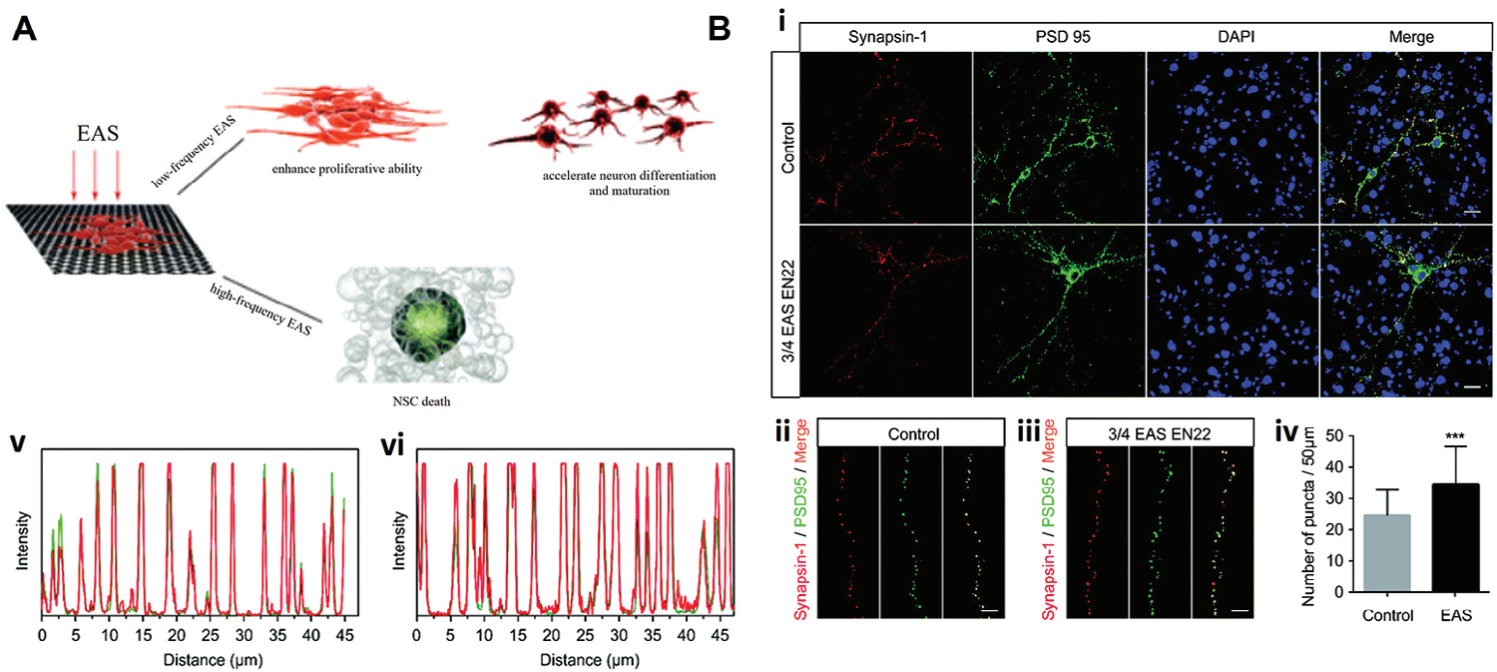
Electrical stimulation promotes stem cell regeneration SGNs.^[40]^ Reproduced with permission. Copyright 2021, Royal Society of Chemistry. A) Electroacoustic stimulation based on cochlear implantation regulates nerve regeneration derived from neural stem cells. B) Low‐frequency EAS accelerated the synaptogenesis of NSC‐derived neurons. i) After low‐frequency EAS stimulation, the representative low‐magnification immunofluorescence images of presynaptic marker anti‐synaptophysin ‐1 and postsynaptic marker anti‐PSD95. Scale bar: 20 mm. ii, iii) High‐magnification fluorescence images of axonal terminal synapse after low‐frequency EAS stimulation (iii) and control (ii). Scale bar: 2 mm. iv) Quantification of the density of the merged synaptic puncta in A. v, vi) Corresponding fluorescence intensity profiles of the merged images in ii and iii, respectively.

As shown in **Figure** **11**, Guo et al. designed a device that can electrically stimulate neural stem cells by combining CIs with graphene substrates.^[^ ^40^ ^]^ In the device, graphene was used as both the substrate of the cell culture system and the reference electrode. The Graphene in this device has been proven to significantly promote the differentiation and maturation of NSCs neurons. It was found that the neurotoxicity of EAS based on CI depends on its unique parameters, such as amplitude, frequency, and duration. These parameters could regulate the survival of NSCs. At the same time, the device can promote the proliferation of neural stem cells and differentiate into neurons under low‐frequency electrical stimulation. However, the acceleration of neuronal differentiation of neural stem cells by low‐frequency electrical stimulation may be the result of the accumulation of various signals, and the specific mechanism of the device needs further study.

Stem cell differentiation is a complex process, which is regulated by many external and internal factors. Compared with chemical or biological‐induced differentiation, electrical stimulation has the advantage of controlling the stimulation precisely and selectively. Therefore, the electrical stimulation applied to promote stem cell regeneration SGNs will have great prospects in the treatment of hearing loss. In the future, more research should be done on the mechanism of 3D scaffold materials and electrical stimulation to promote stem cell differentiation. The combination of new conductive materials and stem cells will contribute to the application of stem cell therapy in hearing loss and even the treatment of neurological diseases.

#### 4.1.3 The Protective Effect on SGNs

Many studies have used cats,^[^ ^17^ ^]^ guinea pigs,^[^ ^100^, ^173^ ^]^ and rats to study the effects of acute and chronic electrical stimulation on nerve response. It is clear that chronic electrical stimulation inside or outside the cochlea has no significant effect on the somatic cell area of mature neurons. It has been found that electrical stimulation in vivo can reduce the death of SGNs after hair cells fall off. Kopelovich et al. directly evaluated the intracellular signal transduction related to apoptosis by monitoring the phosphorylation of nuclear transcription factor Jun by c‐Jun N‐terminal kinase.^[^ ^174^ ^]^ The study found that electrical stimulation can inhibit the known intracellular signal pathway of an apoptosis‐Juk‐Jun pathway. Moreover, the effectiveness of electrical stimulation inhibition depended on the position of the electrode, the pre‐existing phosphorylation level of Jun, the duration of deafness, and the stimulation rate. It also explained why different electrical stimuli have different effects in preventing SGNs death in vivo. The combination of neurotrophic substance delivery to CIs and electrical stimulation can protect SGNs.^[^ ^42^, ^175^ ^]^ Richardson et al. used polypyrrole‐coated electrodes to store neurotrophic factors and 0.1 ng neurotrophic factor could be released every day while giving electrical stimulation.^[^ ^42^ ^]^ After a while, the threshold of eABR in guinea pigs with polypyrrole‐coated electrodes was lower and the density of SGNs in the cochlea was higher. Shepherd et al studied the exogenous delivery system of brain‐derived neurotrophic factor (BDNF) combined with chronic electrical stimulation in guinea pigs with deep deafness.^[^ ^175^ ^]^ It was found that this system has a highly significant nutritional function for SGNs, and the threshold of eABR in guinea pigs was reduced. Combining inner ear therapeutic drugs with chronic electrical stimulation could enhance the protective effect of electrical stimulation on SGNs^43^. Dexamethasone (DEX) can reduce the possible residual hearing loss after cochlear implantation. Scheper et al. combined DEX drug delivery with chronic electrical stimulation.^[^ ^43^ ^]^ It was proved that the use of electrical stimulation can enhance the protection of SGNs in the basal area while drug delivery and the hearing threshold tended to decrease. Therefore, the DEX tends to improve the protection of neurons by increasing the number of surviving neurons.

To sum up, simple electrical stimulation in vivo can prevent the death of SGNs. The combination of neurotrophic factors or inner ear therapeutic drugs with chronic electrical stimulation can enhance the protective effect of electrical stimulation on SGNs, which has great prospects in protecting residual SGNs after cochlear implantation.

### 4.2 Applications of Photostimulation in Promoting SGNs Regeneration

#### 4.2.1 Infrared Nerve Stimulation(INS)

The use of lasers as a method of stimulating neurons was reported as early as 1971.^[^ ^176^ ^]^ Fork et al. used a 488 nm laser to selectively stimulate neurons in the abdominal ganglia of the marine mollusk Aplysia californica.^[^ ^177^ ^]^ The neurons could evoke action potentials at stimulation levels >12.5 mW. The use of laser stimulation applied for the auditory nerve was first reported in 2006. Izzo et al. conducted acute in vivo experiments in gerbils using a holmium yttrium neodymium garnet laser with a wavelength of 2.12 mm, a pulse duration of 250 µs, and an operating frequency of 2 Hz. The compound action potentials induced by cochlear optically (CAPs) were recorded.^[^ ^44^ ^]^ The results showed that optical radiation could induce CAPs in animals. In the absence of inner ear hair cells, the measured stimulation threshold was 0.018 ± 0.003 Jcm^−2^ (mean ±SE). Therefore, it implied that the auditory nerve can be stimulated by light radiation and kicks off the research on INS.

**Figure 12:**
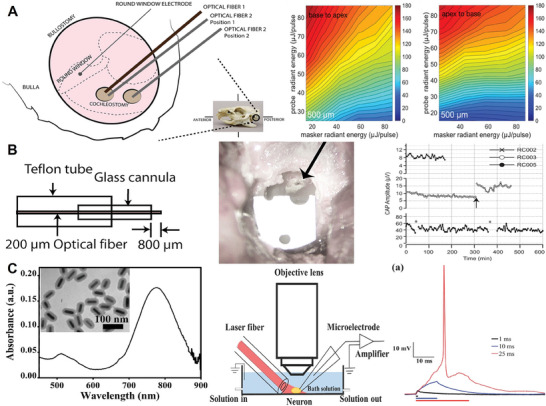
Studies of infrared neural stimulation. A) Experimental protocol for evaluating the stimulation parameters of infrared light stimulation of the cochlear implant on a feline model.^[^ ^178^ ^]^ Reproduced with permission. Copyright 2010, Elsevier. B) Protocol for an experiment to allow quantification of the interaction between adjacent light sources during cochlear stimulation with infrared radiation.^[^ ^181^ ^]^ Reproduced with permission. Copyright 2024, Wiley‐VCH. C) Experimental protocol for near‐infrared stimulation using AuNRs to assist the primary auditory neurons.^[^ ^183^ ^]^ Reproduced with permission. Copyright 2024, Wiley‐VCH.

Tests on the possibility of cochlear damage due to INS have been done on animal models such as guinea pigs^[^ ^45^ ^]^ and cats.^[^ ^178^, ^179^ ^]^ Goyal et al. determined the damage threshold for acute INS in the guinea pig cochlea using compound action potentials and histological assessment.^[^ ^45^ ^]^ The laser parameters for INS include wavelength (*λ* = 1869 nm), pulse duration (100 us), pulse repetition rate (250 Hz), and radiant energy (0–127 uJ pulse^−1^). At a pulse frequency of 250 Hz and radiation energy of up to 25 µJ pulse^−1^, no functional or histological damage was observed after the cochlea was continuously irradiated for up to 5 h. Functional damage to the cochlea occurred only when the energy exceeded 25 µJ pulse^−1^. In another report, Rajguru et al. used a small INS device with an IR wavelength of 1850 nm and a pulse repetition frequency of 200 Hz to stimulate the auditory neurons of five adult cats for up to 10 h and assessed auditory neuron damage by means of CAP amplitude and histologic sectioning (**Figure** **12**A).^[^ ^178^ ^]^ The CAP induced by IR stimulation remained stable and similar in overall shape at the end of the experiment and did not cause any detectable tissue damage to the cochlea. In consequence, IR stimulation was insufficient to produce thermal damage to the tissue to hinder the application of INS for hearing restoration.

Researchers have identified the spatial selectivity of INS for stimulating auditory neurons along the beam path. Moreno et al. used a pulsed infrared laser to stimulate the cochlea of guinea pigs, and the beam path in the cochlea was holistically analyzed by histological reconstruction and micro‐CT scanning of the cochlea.^[^ ^180^ ^]^ The study demonstrated that the neural structures that can be stimulated by INS. It was determined by the optical pathway in the cochlea and the point of incidence on the spiral ganglion. On the other hand, it is important to determine the number of independent channels in the optical cochlear implant based on spatial selectivity. An increase in the number of independent channels facilitated sound perception and music appreciation in noisy environments. As shown in Figure 12B, the forward masking method was used to quantify the interactions between two adjacent light sources during stimulation in seven adult albino guinea pigs.^[^ ^181^ ^]^ The experimental data predicted that the INS‐based optical cochlear implant provided ≈18 independent channels, about twice as many as electrical stimulation, and better improved sound perception in patients. The shortcoming of the study is that, due to the limitations of the cochlear configuration, the research experiments were limited to the basal turn, and the researchers were unable to place the optical fibers in the scalar tympanic chamber at the top of the cochlea. This could have affected the prediction of the number of independent channels.

Whether laser stimulation can provide sufficient temporal resolution to transmit information is also an important area of research. The maximum sustained depolarization rate that can be achieved by a single auditory neuron is 300 to 400 pulses per second (pps). The mean maximum sustained drive rate for optical stimulation was 97 ± 52.5 AP s^−1^, which is similar to the reported maximum sustained acoustic drive rate. Thus, an optical cochlear implant would need to drive auditory neurons at such a rate, or at least at a rate that would achieve normal hearing ideally. In addition, for a range of optical stimulation rates from 2 to 130 pps, the delay jitter time was 300 ms (20 to 40 µs for electrical stimulation). Speech perception can be improved by increasing the delayed jitter time for depolarization of auditory neurons. In conclusion, these studies demonstrated that laser stimulation with appropriate parameters can meet the design requirements for temporal resolution in oCIs.

The use of gold nanorods (AuNRs) in conjunction with infrared stimulation is a new way for researchers to improve the efficiency of the INS and increase the depth of penetration of the INS.^[^ ^182^, ^183^ ^]^ As shown in Figure 12C, Yong et al. used a 780‐nm near‐infrared laser to stimulate primary auditory neurons in culture in rats.^[^ ^183^ ^]^ These neurons were incubated with silica‐coated AuNRs as exogenous absorbers. The researchers used a whole‐cell membrane clamp to observe the compound action potentials of the neurons. Nanorod‐treated auditory neurons (NR‐AN) showed a significant increase in electrical activity compared to the other two experimental groups (neurons co‐incubated with non‐absorbent silica‐coated AuNRs, neurons incubated alone). A transient temperature increase near the surface of the NR‐AN was measured by open pipette electrodes, and the researchers speculated that the laser‐induced heating mechanism of the AuNRs may be related to the previously proposed electrostatic mechanism of membrane capacitance changes.

#### 4.2.2 Optogenetic

The study of optogenetics in hearing restoration includes several aspects, the optogenetic proteins suitable for auditory coding in SGNs, the safe and robust gene delivery of optogenetic proteins to the SGN, and the light generated or introduced to stimulate the SGNs.

The optogenetic proteins that are most suitable for auditory coding in SGNs were studied and used. The encoding of sounds needs very strict temporal accuracy, which requires good temporal responsiveness of the optic proteins applied to SGNs. Eventually, it was chosen to utilize Channelrhodopsin‐2 (ChR2), a light‐gated cation channel from the green alga Chlamydomonas reinhardtii, as a natural photosensitive protein for neuronal light stimulation. Under the irradiation of blue light, mammalian SGNs expressing ChR2 depolarize rapidly. Thus, ChR2 with good photodynamics showed potential for the development of hearing restoration using optogenetics. Nikolic et al. proposed a four‐state model for accurately tracking the photocurrent of ChR2, established parameters more suitable for experimental measurement of the current, and measured the fitting parameter of ChR2 activation time constant is *τ*_ChR_ = 1.3 ms in the case of short stimulation.^[^ ^184^ ^]^ Nevertheless, the sound coding perceived by SGN‐ can reach sub‐millisecond precision as well as peaks of hundreds of Hz and it was experimentally confirmed that the neural activation achieved by ChR2 could not reach the temporal parameters of auditory stimuli. For this reason, ChR2 mutants with faster temporal responses have been continuously developed and a series of research results have been achieved.^[^ ^46^, ^185^, ^186^ ^]^ Afterward, Kleinlogel et al. introduced a new mutant, calcium translocating channelrhodopsin (CatCh).^[^ ^185^ ^]^ This mutant increased the permeability of calcium ions, thereby indirectly affecting changes in membrane potential. It could promote the activation of voltage‐gated sodium channels, and accelerate the response time and voltage response that is ≈70‐fold higher than that of ChR2. Moreover, the fast optic protein Chronos is considered the most promising ChR2 mutant for development. Keppeler et al. effectively transfected Chronos into the cochlea of mice.^[^ ^186^ ^]^ Thereafter, optical stimulation with fiber optics elicited an oABR in mice. Recordings of individual SGNs showed that the light‐evoked spike pulses had good temporal precision. This result demonstrated the feasibility of ultrafast optogenetic stimulation of the auditory pathway by Chronos. As it is known to all, the transition from the closed to the open state is associated with the movement of helix F in microbial‐type retinoblasts. The helix F mutants include the fast mutant Chrimson Y261F/S267M (f‐Chrimson) and the very fast mutant Chrimson K176R/Y261F/S267M (vf‐Chrimson). The Y261F mutation could accelerate the channel closure in Chrimson. Correlation studies^[^ ^46^ ^]^ tested that the closure kinetics of f‐Chrimson ranged from *τ* closure = 24.6 ± 0.9 ms to *τ* closure = 5.7 ± 0.5 ms, while vf‐Chrimson had ultrafast closure kinetics of *τ* closure = 2.7 ± 0.3 ms.

**Figure 13:**
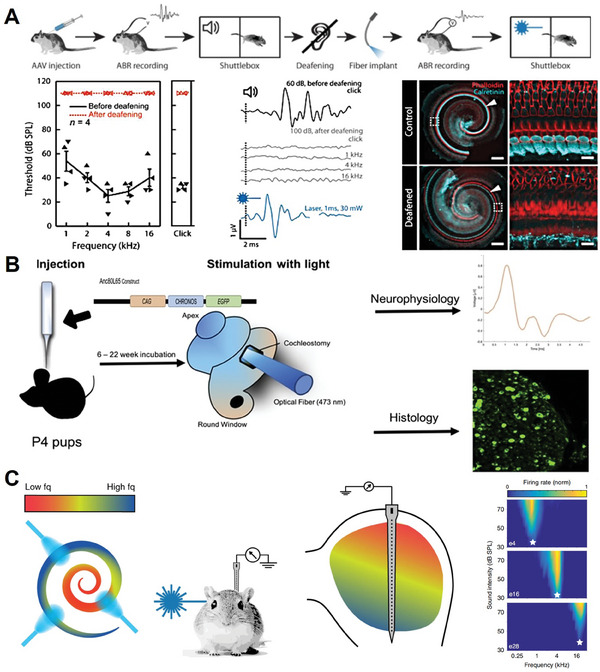
Selected studies of optogenetics in hearing restoration. A) Verification that optogenetic stimulation of cochlear neurons activated the auditory pathway and restored auditory‐driven behaviors in deaf adult gerbils in a deaf adult gerbil animal model.^[^ ^47^ ^]^ Reproduced with permission. Copyright 2024, AAAS. B) Use of ancestral adeno‐associated viral vectors for the delivery of optogenetic proteins to SGNs Experimental protocol.^[^ ^189^ ^]^ Reproduced with permission. Copyright 2018, Elsevier. C) Study of SGNs optogenetic stimulus Experimental protocol for spectral diffusion of excitation.^[^ ^200^ ^]^ Reproduced with permission. Copyright 2019, Springer Nature.

The safe and robust gene delivery of optogenetic proteins to the SGN. In neuroscience, researchers have developed optogenetic‐based optogenetic strategies for optogenetic protein targeting, such as viral promoter targeting, projection targeting, transgenic animal targeting, and spatiotemporal targeting.^[^ ^187^ ^]^ The viral promoter‐targeting strategy is rapidly becoming the most popular research method in the field of auditory research due to its advantages of rapidity in experimental execution, flexibility, genetic and anatomical specificity, high expression potency, and high biosafety. Among the various viruses tested, the performance of the adeno‐associated virus (AAV) applied to the SGNs was the most striking.^[^ ^121^, ^188^, ^189^, ^190^ ^]^ ChR2‐GFP and Halorhodopsin fused with mCherry were successfully transported into the dorsal cochlear nucleus by adeno‐associated viral vectors. They could be successfully transported to the dorsal cochlear nucleus (DCN) and consistently expressed for at least 18 months.^[^ ^190^ ^]^ This approach was shown to enhance the activity of transfected neurons and also demonstrated that the expression of retinoids did not adversely affect hearing. The investigators noted that viral targeting of retinoids could modulate neural pathways and specific neuronal activity and be used as a light beam tracer to map neural pathways. Hernandez et al. delivered Catch to the SGNs of transgenic mice utilizing AAV. It was shown to enhance neural pathway activity by oABR in individual SGNs and in the inferior colliculus (inferior).^[^ ^121^ ^]^ The optogenetic activation of the SGNs was demonstrated by oABR, light‐induced single SGN activity, and Local Field Potential (LFP) recorded in the inferior colliculus (IC). Since then, another study has also successfully transfected AAV‐mediated Catch onto the SGN of adult deaf gerbils and restored the auditory drive behavior of the gerbils (**Figure** **13**A).^[^ ^47^ ^]^ In a study on the efficacy and safety of hearing restoration, Bali et al. injected young mice with AAV2/6 vectors which were loaded with f‐Chrimson, and conducted a longer longitudinal study. The results showed that f‐Chrimson expression in the SGNs persisted for at least 2 years after a single cochlear AAV injection.^[^ ^188^ ^]^ However, f‐Chrimson expression decreased with age. Meanwhile, studies of visceral pathology have shown that localized regions of viral delivery have no effect on visceral organ changes. The more established delivery methods are generally mediated by AAV2/6, which is achieved by injection of ChR2 mutants in early‐birth mice. Nonetheless, current researchers are providing different insights on delivery methods from additional. Anc80L65 is a computerized reconstruction of an AAV vector by researchers with coat proteins close to the ancestral state of AAV serotypes 1, 2, 3, 8, and 9. Compared with previous AAV vectors, Anc80L65 has higher transduction efficiency and stability. The first in vivo experiment in mice with cochlear optogenetics using Anc80L65 mediated delivery of Chronos to the SGN and its activation successfully demonstrated the mediating ability of Anc80L65 (Figure 13B).^[^ ^189^ ^]^ Hernandez et al. used a transuterine pathway in the mouse embryonic ear to express CatCh. It is an approach that has the advantage that several animals can be operated on simultaneously per surgery.^[^ ^121^, ^122^ ^]^ Then, Brigande et al. used an electroporation‐mediated gene transfer approach to deliver genes into the developing mouse inner ear.^[^ ^191^ ^]^ The advantage of this method is that no viral mediation is required. In the future, it may be possible to achieve electroporation through cochlear electrodes, but the difficulty of realizing the technology and the safety and stability of the technology still need further study.

The light needs to be generated inside the cochlea or external light introduced into the cochlea to stimulate the SGNs. This technique of multichannel oCIs is based on two main strategies: active oCIs and passive oCIs. The active oCIs can bring photoelectric emitters into the cochlea and are currently realized mainly by means of miniaturized light‐emitting diodes. Thanks to advances in micro‐nanofabrication technology, cochlear implanted oCIs can incorporate hundreds of micro‐LEDs that can provide the luminescent output power needed to elicit SGNs photostimulation.^[^ ^192^ ^]^ At the same time, the LEDs are positioned close to the SGNs, which avoids the loss of light that occurs when coupling in and out of the waveguide. In the design of active oCIs, attentions still need to be paid to controlling the heat during photostimulation and choosing the right form factor and substrate stiffness. Goßler et al. reported a flexible uLED probe with a width of 230 µm, in which a linear array contains four 50 × 50 µm^2^ uLEDs^193^. By transferring the laser layer from sapphire to polyimide carrier wafers on the silicon transfer process, the prepared GaN substrate probes are ultrathin and highly flexible and can emit up to 60 µW at 1 mA. In addition, the uLEDs in the linear array are contacted via p‐side and n‐side conductive paths, which allows the oCI to handle up to three independent numbers of channels. Klein et al. designed the oCIs to have good thermo‐mechanical properties.^[^ ^194^ ^]^ The researchers used an epoxy resin layer instead of a conventional polyimide (PI) substrate. They also developed a spin‐coating process that allowed the thickness of the epoxy resin layer to vary by <5 µm across the carrier wafer, realizing that up to 144 small light‐emitting diodes (uLEDs) could be integrated into the oCIs probe. Thermal probe characterization showed that the temperature rise of the oCIs can be limited to <1 K with uLEDs powered by DC currents up to 10 mA. When the uLED was operated at 10 mA, 10 kHz, and 10% duty cycle, the optical output power and peak wavelength of the oCI were 0.82 mW and 462 nm, respectively. The optical power corresponded to a radiant emissivity of 407 mW mm^−2^, which meets the requirement of being able to carry out optogenetic experiments. Passive oCIs use a waveguide to deliver the light from the extracochlear emitters into the cochlea.^[^ ^195^ ^]^ Commonly, waveguides are used to introduce light from an external light source through an invasive interface to the target tissue for the appropriate optogenetic experiments or near‐infrared photostimulation. The main advantages of this approach are less invasive to the tissues, less thermal damage to the target tissues, a high degree of device integration, good stability, and so on. However, there is a certain optical loss in the emitter‐waveguide interface and the internal coupling process along the waveguide. This approach has not yet been exploited maturely in the field of oCIs, and the probes for optical waveguides are mainly designed for brain applications.^[^ ^196^ ^]^ Alt et al. summarized the implantable neural probe technologies developed in recent years.^[^ ^196^ ^]^ The optical probes were summarized from the perspectives of substrate materials, invasive interface design and requirements, and light sources, providing fundamental insights for researchers to design passive oCIs in the future. Balster et al. verified the feasibility of implanting optical fibers into the cochlea with minimal trauma.^[^ ^197^ ^]^ The researchers inserted glass fibers with different inner core/cladding diameters into a human tympanic membrane model, and ST model and performed insertion force measurements on the cochlear implant model. Thinner glass fibers that showed low insertion force in the model were then inserted into the human temporal bone. The results showed that the finer fibers (core/cladding diameters of 20/25 µm) could be inserted into the model without breaking and could be inserted into the human temporal bone without traumatizing the inner ear structures.

Meanwhile, there are still some ways to go before oCIs based on optogenetics are actually used in clinical care. Researchers need to further optimize and adjust its parameters in terms of safety testing (biocompatibility, long‐term use safety, and stability), temporal and spatial resolution, and dynamic range.

Safety testing. In the study of transfecting ChR visual proteins onto SGNs in mammals, it has already been shown that the ChR visual proteins have no damage to hearing in animal models.^[^ ^190^ ^]^ In order to improve the temporal resolution of sound encoding, it will be inevitable to see continued research and use of ChR2 mutants with faster dynamics in the future. The biosafety assessment of these mutants is a critical step in research and cannot be ignored. One of the reasons that researchers typically use ChR2 mutants with red‐shifted effects for transfection is that red light radiation is less phototoxic to the cochlea when photostimulation is performed in the cochlea. This approach also means that higher‐powered red light can be used for stimulation without triggering adverse tissue reactions. Senova et al. used pulsed red light with irradiances ranging from 100 to 600 mW mm^−2^ to photostimulated deep cortical brain tissue by applying 5 ms pulses of 20, 40, and 60 Hz over 90 s.^[^ ^198^ ^]^ in vivo electrophysiological recordings and histological analyses showed that high‐power photostimulation had no significant phototoxic effects and did not trigger nonphysiologically functional activation. Meanwhile, oCIs require long‐term implantation in animal models. Assessing the safety and stability of long‐term implants is also a very important step. In a study by Keppele,^[^ ^199^ ^]^ an integrated Pt bender was first used to characterize heat generation and heat dissipation. The results showed that the scheme used for worst‐case estimation (100% DC, current intensity = 38.9 mA, current pulse = 10 ms) exceeded the feasibility of in vivo stimulation. Afterward, in the absence of silica gel for encapsulation, the researchers performed lifetime tests on oCIs treated with different encapsulation materials to confirm the stability of uLEDs for long‐term use. The results showed that the combined use of repetitive plasma treatment technology, epoxy bottom filler, and Cytop encapsulation technology could extend the lifetime of oCIs to >200 days. Moreover, the final encapsulation of the oCIs with silica gel can further support mechanical stability and extend the lifetime. It is owing that the encapsulation can prevent water vapor from penetrating into the photoelectric emitter and causing damage to the device.

Temporal selectivity. Researchers have assessed the spatial resolution of oCIs by recording the electrophysiological activity of the central nucleus of the IC (ICC) in the auditory midbrain of mice. ICC electrophysiological activity is characterized by high spatial fidelity to the stimulus that triggers the auditory response.^[^ ^121^ ^]^Thus, assessment of ICC neuronal activation can directly infer the degree of excitation diffusion within the cochlea. The study by Keppele et al. measured the excitation spread of LED‐based oCIs (1.9/2.4/3.4/4.1 octave, d′of 1.5/2/2/2.5/3). They are better than those of monopolar and bipolar eCI stimuli (1.6/3.6/6.5/6.9 and 2.2/4.2/6.2/6.9 octave, respectively), confirming that optogenetics can lead to an increase in the spatial resolution of CI sound coding.^[^ ^199^ ^]^ Similar findings were obtained in another study.^[^ ^167^ ^]^ Four neighboring uLEDs with a pitch of 100 to 150 um were used. The average spatial extensions of excitations in the ICC under SGNs photostimulation were 0.95/1.93/2.94/3.06 octave, respectively, when d′at BE was 1.5/2/2.5/3 (individual uLEDs) Furthermore, as shown in Figure 13C, researchers performed multichannel recordings of neuronal clusters in the phonotactically organized central nucleus of the Mongolian gerbil IC by optogenetic and electrical stimulation of the SGNs to compare the spatial selectivity of the two devices.^[^ ^200^ ^]^ The results showed that the spatial selectivity of optogenetic stimulation was superior to that of electrical stimulation in most cases. In a nutshell, these studies further demonstrate the superior spatial selectivity exhibited by optogenetics. The temporal properties of optogenetic activation of the SGNs depended largely on the kinetics of the optic proteins. Researchers typically characterized the temporal properties by recording oABR and single SGN electrophysiological activity. oABR can be used as an estimator of temporal precision because signal amplitude varies with the number of neurons and the degree of synchronization.

Dynamic range. Normal hearing has a dynamic range of up to 120 dB, but the output dynamic range of eCI coding is limited to 10–20 dB.^[^ ^201^ ^]^ The synergistic effect of traveling wave amplification by outer hair cells and various other physiological mechanisms endows the cochlea with the diversity of sound coding at different locations in the cochlea. Due to the lack of such physiological mechanisms, whether electrical or optical stimulation, the cochlear implant can provide a dynamic range that is much smaller than the normal values provided by physiological structures. Researchers can maximize optogenetic stimulation to extend the dynamic range in the future by maximizing power output, optimizing transmitter placement to increase irradiance to the SGNs, and increasing the photosensitivity of ChR2 mutants transfected with SGNs^202^.

### 4.3 Applications of Magnetic Stimulation in Promoting SGNs Regeneration

Magnetic‐responsive neuromodulation is an important neuromodulation strategy in the future.^[^ ^203^, ^204^ ^]^ This section summarized the existing works applied to SGNs and provided an outlook for future research on SGNs from the perspective of the advances in neural regeneration and neuromodulation. Currently, there are fewer reports on the effects of magnetic stimulation on SGNs, but most of them are focused on nerve regeneration. Hu et al. investigated the changes in neurite growth and orientation after direct uptake of superparamagnetic iron oxide (SPIO) by SGNs^[^ ^48^ ^]^ in vitro. Chai's group utilized the self‐assembly of magnetic colloidal nanoparticles to form nanoclusters in the presence of an MF.^[^ ^49^ ^]^ Then, the potential relationship between SGNs and nanoclusters was investigated. The study revealed the potential mechanisms by which nanoclusters act on SGNs in terms of cell adhesion, guidance of neurite growth, growth cone development, and the effects of synaptogenesis. These properties enable magnetic colloidal nanoclusters to be a versatile research platform in vitro for the directional alignment of SGNs in the future.

#### 4.3.1 Nerve Regeneration

##### Direct Uptake of SPION

**Figure 14:**
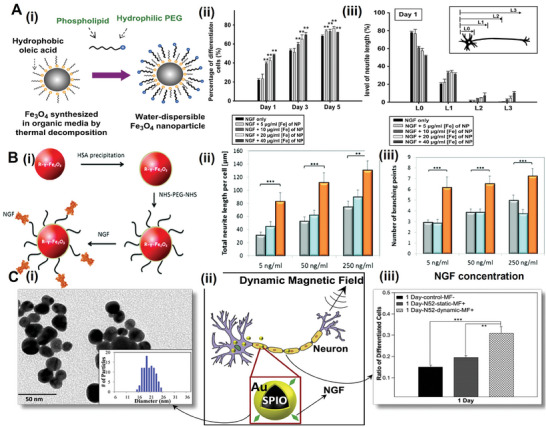
Direct uptake of SPION by PC12 cells. A) i) Schematic representation of the synthesis of iron oxide nanoparticles.^[^ ^131^ ^]^ ii) Effect of iron oxide nanoparticles on differentiation efficiency with respect to dose and time. iii) Distribution of neurite length in PC12 cells 1 day after induced differentiation. Reproduced with permission. Copyright 2011, Elsevier. B) i) Schematic diagram of NGF‐NPs synthesis.^[^ ^124^ ^]^ ii) The influence of NGF‐NPs on morphological parameters of neuronal differentiation at different NGF conceptions. iii) Number of branch points. Reproduced with permission. Copyright 2009, Royal Society of Chemistry. C) i) TEM image of prepared SPIO‐Au NPs (20.8 nm).^[^ ^135^ ^]^ ii) Schematic representation of the action of SPIO‐Au NPs iii) PC‐12 cells after dynamic MF treatment and without dynamic MF treatment. Percentage of differentiated neuronal phenotype cells. Reproduced with permission. Copyright 2018, Elsevier.

The use of SPION in combination with Nerve Growth Factor (NGF) has been studied for more than a decade. Initially, Kim et al. added iron oxide nanoparticles together with free NGFs to the culture medium of PC12 cells (**Figure** **14**A).^[^ ^131^ ^]^ By comparing it to an experimental group of PC12 media treated with NGFs alone, the researchers verified that iron oxide nanoparticles could promote the effects of NGFs on PC12 neurite growth. Furthermore, researchers have continued to improve the materials in order to achieve better therapeutic effects. As shown in Figure 14B, Marcus et al. covalently coupled NGFs on iron oxide nanoparticles encapsulated with PEG.^[^ ^124^ ^]^ The novel NP‐NGF composite could be used to slow down the degradation rate of NGFs, allowing for a further increase in therapeutic efficacy on the growth of PC12 neuromasts. When iron oxide nanoparticles were hydrolyzed to iron ions in cells at a high concentration, the generation of reactive oxygen species (ROS) led to the potential cytotoxicity of MNPs^205^. It reported that a variety of naturally occurring flavonoids with antioxidant activity can reduce the toxicity of ROS in neuronal cells. Therefore, Katebi and his cooperators used quercetin as an important neuroprotective agent by combining it with SPIONs and NGFs to enhance the role of NGFs during neural differentiation of cells.^[^ ^206^ ^]^ The results showed that this regimen did further promote neuronal branching morphogenesis in PC12 cells. Meanwhile, one of the important features of iron oxide nanoparticles is their ability to synergize with MFs. For this reason, as shown in Figure 14C, Yuan et al. investigated the effect of NGF‐functionalized iron‐gold oxide nanoparticles on neurite elongation and orientation in PC12 with a dynamic MF.^[^ ^135^ ^]^ This study confirmed that dynamic MFs significantly enhanced neurite growth and preferentially aligned neurites to grow along the direction of MF. These studies were instructive for the applications of iron oxide nanoparticles to SGNs. In addition, Chai's group prepared SPIONs by chemical co‐precipitation with narrow particle size distribution, low cytotoxicity, and superparamagnetism.^[^ ^48^ ^]^ In the presence or absence of external MF, SGNs could internalize SPIONs. The neurite extension of SGNs was promoted after internalization. It was also demonstrated that SPIONs could regulate cell migration. SPIONs could preferentially guide the growth of neural synapses in SGNs along the direction imposed by external MF. These findings provided fundamental insights into the regulation of cell behavior in response to magnetic cues and suggested new therapeutic directions for SNHL caused by the degeneration of SGNs.

##### Creating a Magnetically Responsive Bionic Extracellular Matrix

**Figure 15:**
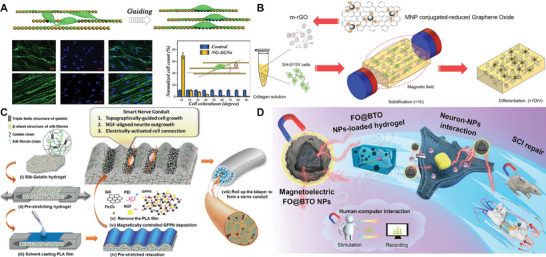
Selected strategies for creating biomimetic extracellular matrices with magnetic response. A) Using magnetic colloidal nanoribbons to guide neuronal synaptic growth.^[^ ^49^ ^]^ Reproduced with permission. Copyright 2022, Elsevier. B) A magnetic nanoparticle‐modified reduced graphene oxide/collagen 3D nanocomposite hydrogel scaffold that promotes neuronal differentiation and guides directional cell growth.^[^ ^125^ ^]^ Reproduced with permission. Copyright CC BY‐NC‐ND 4.0. C) Micropatterning of reduced graphene oxide (rGO), polyethyleneimine (PEI), and iron oxide (Fe_3_O_4_) on SG hydrogels, termed as GFPN complex.^[^ ^213^ ^]^ Reproduced with permission. Copyright 2020, American Chemical Society. D) Schematic diagram of the magneto‐electro‐Fe_3_O_4_@BTO_3_ nanoparticle‐induced tele‐electro‐stimulation for regulating neurogenesis and SCI repair and providing open insights into future applications in the field of human‐computer interaction.^[^ ^214^ ^]^ Reproduced with permission. Copyright 2021, Wiley‐VCH.

The applications of magnetic nanomaterials to neural regeneration of SGN is in its infancy, and yet shows promise. Chai's team innovatively applied magnetic colloidal nanoparticles to the controlled growth of SGNs (**Figure** **15**A).^[^ ^49^ ^]^ These magnetic colloidal nanoparticles consisted of ferrous oxide cores and silica shells covered with a matrix gel coating. The coating was used to increase the nanoparticles' cellular adhesion, biocompatibility, and so on. These MNPs could be self‐assembled under the external MF and segmented to form nano‐chains, which constituted the complex network structure required for nerve growth. Through morphological analysis of SGNs grown on the nano‐chains (NG‐SGNs), ≈70% of the NG‐SGNs had an orientation angle of 3° or less with the nano‐chains, which indicated that the nano‐chains have a good guided orientation property. Meanwhile, the dendritic complexity index (DCI) indicated that the dendritic complexity of the NG‐SGNs group was significantly enhanced under the guidance of nanostrands compared with the control group. In order to show the value of nanostrands for neural network reconstruction in SGNs, the team further investigated the growth cone development, synaptic dot density, and calcium ion imaging. All the findings reflected the potential value of magnetic colloidal nanostrands in the field of neural regeneration. The article illustrated the guidance mechanism of magnetic colloidal nanoclusters on primary SGNs at the transcriptome level for the first time, laying the foundation for magnetic colloidal nanoclusters in biomedicine.

MNPs respond well to external MFs. By doping MNPs at the material preparation stage in response to external magnetic field stimulation, the material can be made to form microstructures with well‐defined orientations, which provide topographical cues for the growth of newborn neural synapses. In the current work, the researchers formed a hydrogel system with a certain internal orientation by doping short fibers such as collagen fibers,^[^ ^207^, ^208^ ^]^ electrostatically spun materials,^[^ ^209^, ^210^ ^]^ graphene oxide,^[^ ^125^ ^]^ and microhydrogels^211^bound to MNPs, and using a magnetic field to guide the directional alignment of the short fibers prior to the curing of the hydrogel. In the preparation of collagen hydrogels with a specific orientation, the methods of “magnetic particle string”^[^ ^207^ ^]^ and “magnetic anchor”^[^ ^208^ ^]^ were used. The fabrication of “Magnetic particle stringing” involves mixing MNPs into a collagen hydrogel and then applying an external MF. During gelation, the magnetic particles aggregated into strings, leading to the directional alignment of collagen fibers. In contrast, “magnetic anchoring” refers to the targeted binding of gold magnetic nanoparticles (GMNP) to collagen fibers by coating GMNP with collagen mimetic peptides (CMP). Through the intervention of an external MF, GMNP aligned the collagen fibers in a certain orientation. Neurons grown in the environments constructed by both methods showed normal electrical activity and vigor, and the newborn synapses were more elongated and exhibited good orientation precision. It has been shown that a concentration of MNPs of ≈1.5 mM could reduce the viability of sensitive cells.^[^ ^137^ ^]^ The study related to the “magnetic particle string” method^[^ ^207^ ^]^ clearly used a higher concentration of MNPs (0.05 mg of MNPs per 100 µL of collagen solution), while the “magnetic anchor” method^[^ ^208^ ^]^ used a higher concentration of MNPs (0.05 mg of MNPs per 100 µL of collagen solution). Moreover, a much smaller number of nanoparticles (0.005 mg MNPs per 100 µL of collagen solution) was used in the “magnetic anchor” method.^[^ ^175^ ^]^ Reduced‐state graphene oxide platforms were widely used in the field of nerve regeneration due to their good electrical conductivity and biocompatibility.^[^ ^125^, ^212^ ^]^ Santhosh et al. developed a composite hydrogel^[^ ^125^ ^]^ in Figure 15B, in which reduced graphene oxide modified by MNPs was oriented and aligned. It not only provided spatial guidance cues for the hydrogel‐doped neuroblastoma cells SH‐SY5Y to induce unidirectional growth but also promoted their differentiation into and formation of neurons.

Utilizing the magnetoelectric synergy effect to build a bionic platform for nerve regeneration is also a developmental direction. Lin et al. built a bionic platform with cues of morphology, electrical stimulation, and chemical modulation (Figure 15C).^[^ ^213^ ^]^ The platform used strain‐induced morphology based on the mechanical mismatch between poly lactic acid (PLA) and silk/gelatin (SG) hydrogels to realize ordered ripple patterns on SG hydrogels. The GFPN complexes consisting of reduced graphene oxide (rGO), polyethyleneimine (PEI), and iron oxide (Fe_3_O_4_) were further utilized for micropatterning on SG hydrogels. The method was realized to provide morphological, biological, and electrical stimulation to neurons on the graphene‐based interfaces. Cultured neuronal cells exhibited distinctly ordered arrangement and elongation of neurites. Neurons could achieve further interconnections on the material. As shown in Figure 15D, Zhang et al. utilized a magneto‐electric nanoparticle to influence neuronal behavior by remotely and wirelessly delivering electrical stimulation via an external MF.^[^ ^214^ ^]^ The researchers synthesized the NPs of Fe_3_O_4_@BTO_3_ with a magnetoelectric effect and a core‐shell structure through a sol‐gel process and hydrothermal reaction. They were then loaded into a hyaluronic acid/collagen composite hydrogel (HA/Col). Under this system, the researchers probed the expression of neural functions in PC12 cells, including dendritic morphology, elongated axons, and potential synapse formation. At the same time, the researchers demonstrated that the magneto‐electric response stimulated the neuronal cells and triggered the up‐regulation of L‐VGCC, which resulted in more Ca^2+^ release and ultimately enhanced the neural functional expression of PC12 cells. Finally, the researchers radio‐stimulated spinal cord injury (SCI) rats via a magneto‐electric response and found that the magneto‐electric nanoparticles could improve the recovery rate of SCI.

In addition, the protocol of altering the growth microenvironment of neuronal by doping MNPs has been tested in relevant animal models. Bhattacharyya et al. prepared a gelatin‐genistein hydrogel system containing oleo‐iron‐based superparamagnetic nanoparticles (IONPs) and injected them in the lesion site of a rat model of traumatic SCI.^[^ ^215^ ^]^ It showed significant improvements in behavioral, electrophysiological, and morphological parameters. This study revealed that the deep‐rooted mechanism of IONP recovery in SCI rats was the alteration of neurotrophic factor levels, reduction of activated microglia, and increase of GAP‐43 expression after IONP treatment. These results demonstrate the potential of implanting IONPs embedded in a gelatin‐genistein hydrogel system as well as exposure to MF environments to modulate the microenvironment in nerve repair and regeneration. In another study, researchers implanted IONPs (3 µg mL^−1^) embedded in a sugar gel at the site of injury (13 segments for spinal cord transection) in adult male Wistar rats exposed to MF (50 Hz, 17.96 µT, 2 h per day for 5 weeks).^[^ ^216^ ^]^ Histological analysis revealed a significant increase in the expression of the growth‐related protein GAP‐43. However, the researchers did not observe motor or somatosensory evoked potential responses. This suggested that long‐distance functional connectivity between injured neurons is still lacking. These findings also emphasized the therapeutic potential of IONPs and MF in promoting nerve regeneration after SCI.

#### 4.3.2 Neuromodulation

**Figure 16:**
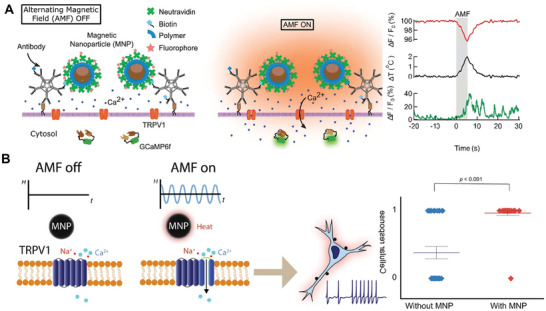
Selected applications of magneto‐thermal neuromodulation techniques. A) Magneto‐thermal genetic neural stimulation activated TPRV1 channels by using alternating MF heated membranes in combination with MNPs.^[^ ^218^ ^]^ Reproduced with permission. Copyright CC BY‐NC‐ND 4.0. B) MF stimulation (“AMF ON”) induces MNP heating, which induces membrane depolarisation.^[^ ^219^ ^]^ Reproduced with permission. Copyright 2021, Springer Nature.

The earliest report on the use of magnetic nanoparticle‐based radiofrequency MFs to remotely activate temperature‐sensitive ion channels to modulate neurons via magnetogenerated heat was proposed in 2010. Huang et al. designed a superparamagnetic nanoparticle.^[^ ^217^ ^]^ This SPION could target TRPV1 channels to stimulate specific cells and achieve a high‐density enrichment near the plasma membrane, which resulted in localized heating of the plasma membrane without affecting the overall temperature. The TRPV1 channel is a temperature‐sensitive channel with an activation temperature of 42 °C (close to the normal body temperature), allowing for rapid stimulation and channel normoclosure. The team induced the generation of action potentials on hippocampal neurons cultured in vitro without damage to the neurons. In the behavioral response experiments of live animals, the researchers targeted all sensory neurons in the amphibious region of the hidrobatid nematode with nanoparticles and initiated a thermal avoidance response in the hidrobatid nematode by applying a radiofrequency MF. These studies are sufficient to show that magnetic stimulation is a very promising remote and non‐invasive modality for neuromodulation. After years of development, magneto‐thermal neuromodulation is well used in deep brain stimulation (DBS). Munshi's group successfully activated specific neural circuits in behaviorally autonomous, awake mice using magneto‐thermal neuromodulation (**Figure** **16**A).^[^ ^218^ ^]^ The mice were able to respond to motor cortex stimulation and thus perform the appropriate movements‐rotating around the body axis after stimulation deep in the striatum, freezing gait in response to stimulation of the dorsal and ventral interstriatal ridges. Afterward, as shown in Figure 16B, Hescham et al. utilized magnetothermal nanoparticles in mice with Parkinson's disease after administering magnetothermal deep brain stimulation (mDBS) and then observed the treatment effect.^[^ ^219^ ^]^ The results showed that mDBS of the subthalamic nucleus (STN) reversed motor deficits in mild and severe Parkinson's disease models. The application of magneto‐thermal neuromodulation technology in DBS fully demonstrated its properties of high sensitivity, high targeting, and non‐implantable. It predicted that the magneto‐thermal neuromodulation technology has great prospects for future development in the neuromodulation of SGNs. Application of physical stimulation in promoting SGNs regeneration was provided in **Table** **2**. The different strategies were studied, and the different effects were shown. The aim is to provide theoretical and experimental basis for the development of novel electrical stimulation therapy strategies.

**Table 2:** Summary of the representative work of existing physical stimulation in promoting SGNs regeneration.

Physical Method	Materials/equipment	Stimulus parameter	Effect	Refs.
Electrostimulation	CI/graphene	pulse width: 25µs, the amplitude of the T‐level: 138CL, the amplitude of the C‐level: 185CL.	Promote the expression levels of proliferation‐related genes and enhance the activity of neurons to express synaptic proteins.	[40]
	Ti_3_C_2_T_X_ Mxene	pulse width: 25µs, amplitude of the T‐ level: 138CL, the amplitude of the C‐ level: 185CL. 7 days, 10min every day.	The growth cone area and filiform foot are larger, the number of synapses is increased, and the frequency of calcium oscillation is increased.	[39]
	CI/graphene	pulse width: 25µs, amplitude of the T‐ level: 138CL, amplitude of the C‐ level: 185CL. current: 0.21 mA and 0.49 mA 5 days, 10 h every day.	Promote the development of neurites, growth cones, and filamentous feet of SGNs.	[144]
Photostimulation	Diode laser	wavelength: 1.92 – 1.94 µm, pulse duration: 5 – 300 µs laser repetition frequency: 2 Hz	efficient delivery of stimulus energy to neurons	[44]
	50 µm fiber‐coupled 594 nm laser	pulse width: 3 ms, wavelength: 594nm, saturation intensity: 11–30mW mm^−2^	Achieved efficient delivery and expression of fast Chrimson mutants; enabled SGNs with near‐physiological spike rates and spike times; restored auditory activity in deaf mice.	[46]
	fiber optically coupled to a blue laser	optical intensity: 0.9‐32.2 mW, stimulation frequency: 10–250 Hz, maximum light pulse: 300 µJ, light intensity: 0.1‐25 mW	Achieved AAV‐mediated CatCh expression in the cochlear SGN of adult Mongolian gerbils; optogenetic stimulation effects; confirmed acoustic perception (animal model).	[47]
	silica‐coated Au NRs and Au NSs	peak laser power: 90 mW, wavelength: 780 nm	stimulate electrical activity in auditory neurons.	[183]
	optical fiber: 200 µm in diameter blue laser: 473 nm, 100 mW DPSS	1 ms laser pulse at maximum stimulus intensity (≈35 mW, 500 µA)	Multichannel recordings in the IC of gerbils show that the spectral selectivity of optogenetic stimulation is superior to that of electrical stimulation SGN.	[200]
Magnetic stimulation	SPIO NPs (core: γ‐Fe_2_O_3_, external coating: PSC)	External static MF: 20–30 mT/50–60 mT/80–90 mT	Regulates the extension and orientation of SGN neuromasts; promotes the development of growth cones; affects cell migration capacity.	[48]
	Magnetic colloidal nanoparticles Inner core: ferrous oxide External Coating: Silicon Dioxide	Static MF: 20 mT	Directing SGN synapse growth orientation, growth cone development, and synapse formation; directing neuronal network formation and signaling communication in SGNs.	[49]

## 5 Challenges and Prospects

### 5.1 Drug Therapy

The existing problems of local drug delivery mainly include poor drug targeting and unclear pharmacokinetics in the inner ear, high invasiveness to tissues, and few clinical studies. Safely increasing the effective concentration of drugs in the inner ear and realizing drug delivery are the key and difficult points of current research. Therefore, several strategies can be adopted to address the existing problems as follows.

1) The development of novel materials.

The development of drug delivery carriers focuses on improving drug targeting, system biosafety, and low damage to tissues in future. Therefore, consideration should be given to endowing carrier materials with more characteristics, such as responding to physical regulatory factors to achieve controlled drug release, surface functionalization to modify antibodies and targeting peptides to improve drug targeting, using new biodegradable biomaterials to improve the system biosafety; and exploring cellular micro‐drug delivery systems to reduce tissue damage.

2) Optimize the existing materials.

Currently, researchers have developed injectable hydrogels, microgels, liposomes, and other drug‐delivery carriers. These carriers have the advantages of high biosafety and low tissue invasiveness, but they also have obvious drawbacks such as low targeting and non‐biodegradability. It is more feasible to optimize the existing materials by giving the materials more characteristics such as high biosafety, high targeting, low tissue invasiveness, and biodegradability.

3) Combination with other hearing therapy strategies.

In recent years, researchers have not only focused on the use of CIs to replace missing hair cells to achieve stimulation of SGNs but also on the modification of cochlear coatings in an attempt to provide regenerative and protective support for SGNs. By loading related drugs into the coating of cochlear implant, the drugs can directly act on the targets in the inner ear to achieve therapeutic effects on hair cells and SGNs. In addition, hearing aids are more widely used to assist hearing function, and their use in conjunction with medication is more universal. Integration of implantable delivery systems with hearing aids will be a new direction in the development of hearing restoration strategies.

### 5.2 Hearing AIDS and CIs

Although hearing AIDS and CIs have been used in practical applications, they still have some problems, such as high price, large size, not widely accepted, insertion trauma, and current diffusion in CIs. Developing new practical materials or using cheap materials such as Polyethylene (PE), using micro‐nano processing technology, and speeding up the research of optical CIs will be conducive to manufacturing miniaturized, cheap, and widely used hearing AIDS or CIs in the future.

Though the conductive materials with different structures combined with electroacoustic systems have good effects on the growth of SGNs, different customized needs of patients and expensive materials are major obstacles to its commercialization. With the development of emerging manufacturing technologies such as 3D printing, this problem may be solved.

### 5.3 Electrostimulation

At present, there are some problems with electrical stimulation. The single physical stimulation makes it difficult to promote complete nerve regeneration and the existing biomaterials have poor conductivity to cells. The materials have poor biological safety and cannot be completely degraded. Furthermore, the optimal parameters and action mechanism of electrical stimulation are unclear. Therefore, it is urgent to innovate the means of electric stimulation, combine various physical stimulation means, innovate materials, optimize stimulation parameters, and clarify the mechanism of action.

1) Innovative means of electrical stimulation

Because the regeneration and functional recovery of SGNs is a long and complicated process, it is difficult to achieve complete nerve regeneration by single electrical, magnetic, and optical stimulation. Therefore, the ultimate goal may be achieved by combining various physical stimuli. For example, the piezoelectric nanobeam with a linear arrangement of polycaprolactone (PCL) and polyvinylidene fluoride (PVDF) made by electrostatic spinning technology can convert external mechanical force into electrical stimulation by applying low‐intensity pulsed ultrasound. Using NGF as a biological stimulus and low‐frequency pulsed ultrasound (US) as the physical stimulus, the release of NGF controlled by low‐frequency pulsed ultrasound is expected to realize the regeneration of SGNs. 1‐methyl‐3‐isobutyl xanthine (IBMX) can be used as a chemical stimulus and mechanical stress can be applied to stimulate stem cells. Because chemical stimulus plays a decisive role in the differentiation of stem cells, physical stimulus can affect the early differentiation of stem cells. Therefore, the combination of chemical stimulation and physical stimulation is expected to promote the differentiation of stem cells into SGNs.

At the same time, for the regeneration of neurons, accuracy, and safety are lacking under direct physical stimulation in vivo. It cannot accurately stimulate cells without affecting surrounding neurons. Therefore, by applying energy to functional materials in a wireless way, the functional materials can stimulate neurons accurately and indirectly It will have a great impact on SGNs and even the regeneration of neurons.

2) The innovation of material design

Based on the conductivity of nerve cells, the development of conductive biomaterials is of great significance for nerve regeneration. Piezoelectric materials may have broad prospects in the treatment of nerve injury. Piezoelectric materials can provide electrical stimulation without invasion and can be combined with other substances to control electrical stimulation remotely. This non‐invasive stimulation method is beneficial to nerve regeneration. In the future, a piezoelectric material system can be applied to CIs, which can not only realize electrical stimulation but also promote the regeneration of damaged SGNs.

Because the repair of SGNs is a long and complicated process, the materials used must be effective in vivo for a long time and have high biological safety. At the same time, it should be noted that biodegradable materials are also necessary for long‐term treatment. At present, a completely bioabsorbable stimulator has been developed, which has become a powerful tool for nerve regulation and regeneration. Their biodegradation rate can be changed by changing the composition of materials, which can realize controllable stimulation on demand. Therefore, we can develop an ultra‐miniature SGN stimulator that is biodegradable and self‐powered. It can accelerate the regeneration of neurons without secondary operation.

In addition, because of the anisotropy of the nervous system, newborn neurons need directional growth to establish connections with synapses of partner cells to play their functions. At present, many materials have been used to promote the directional growth of nerve cells, such as magnetic nano‐materials, fibers, super‐aligned carbon nanotubes, and so on. Some good results have been achieved. Therefore, the applications of cell‐oriented growth‐promoting materials in SGNs regeneration and other fields can promote its clinical applications.

3) Optimizing the electrical stimulation parameters

Excitement transmission in the nervous system is a complex process. It is of great significance to optimize the electrical stimulation conditions that match the electrophysiological characteristics of SGNs, such as voltage, frequency, and stimulation duration. For SGNs, the range of adjustable electrical stimulation parameters may be relatively narrow, but small changes in stimulation parameters will lead to different cell reactions. In the future, the best stimulation parameters of electrical stimulation of SGNs should be determined to achieve the best research and treatment results.

4) Clear mechanism of action

The exact mechanisms of interaction and feedback between SGNs and conductive materials, intracellular signal pathways, and so on are still unclear. At present, it is necessary to focus on revealing the interaction between SGNs and conductive materials, so as to determine the general design principles of electroactive biomaterials and electrical stimulation at the cellular, molecular, and genetic levels of SGNs.

### 5.4 Infrared Nerve Stimulation

1) Clarify the mechanism of action.

The effectiveness and mechanism of action of the photo‐thermal effect and photo‐mechanical effect in infrared stimulation are still unclear. There is an urgent need for researchers to come up with a clear mechanism to guide the targeted applications of INS‐based oCIs for various hearing loss pathologies.

2) Optimization of stimulation parameters

Currently, the research on parameter optimization still remains at the stage of animal experiments, and the parameters of infrared laser and optical fiber used in different studies are largely different. The research status is not conducive to the integration of core devices such as infrared laser generators and optical fibers for clinical trials or mass production. Therefore, researchers still need to optimize the parameters at this stage and unify the best parameters in the animal models for further research.

3) Integration of devices

From confirming the effectiveness of INS for hearing restoration to realizing the core device integration of oCIs, in‐depth and long‐range research is still needed. Taking the more mature electrical stimulation cochlear implant as an example, the programming of the language processor and the implementation of digital‐to‐analog information conversion are all problems that need to be solved for oCIs.

### 5.5 Optogenetics applied to Neuromodulation of SGNs

1) Optimizing retinoids

The exploration of ChR2 mutants with faster photodynamics is a long‐term fundamental study. The temporal resolution that an optical cochlear implant can improve is largely determined by the temporal properties of the optic proteins transfected onto SGNs. Therefore, in the development of oCIs based on optogenetics, researchers can explore the construction of ChR2 mutants from the perspective of gene mutation or gene editing.

2) Construction of safe and stable retinoid vectors

The current adeno‐associated viral vectors used to deliver optic proteins still have the possibility of off‐targeting, resulting in poor transfection of SGNs. Second, the therapeutic effect will be greatly affected by patients who have been infected with AAV. Finally, AAV may accumulate in the vital organs of the human, such as the brain and liver, which will bring potential toxicity. For this reason, it is essential to continuously develop safer and more stable AAV vectors and explore novel retinoid delivery strategies. The selection of a suitable promoter is one of the key factors in designing safe AAV vectors. Re‐expression of AAV‐carrying retinoid genes using promoters with SGNs specificity can reduce the expression of AAV in organs other than the cochlea and decrease the potential toxicity. The development of AAV vectors with high specificity, high stability, and the ability to accommodate large segments of genes through directed evolution, computer bioinformatics, and other methods is one of the most promising directions for future development.

3) Optimization of light‐emitting/transmitting optical devices

For active oCIs that can generate light directly in the cochlea, researchers should focus on optimizing the performance of the devices in the future. The designed oCIs need to provide sufficient light irradiance to meet the requirements of cochlear temporal and spatial resolution. In addition, evaluation indexes for optical cochlear implant performance need to be established and standardized to move away from the evaluation system based on traditional electrical stimulation cochlear implants. As for the design of passive oCIs, researchers can draw inspiration from the implantable neural probe technology that has been widely used for brain stimulation.

### 5.6 The Combination of Physical Stimulation and Other Listening Therapy Strategies

Stem cell therapy and gene therapy are effective treatment strategies for sensorineural deafness. Stem cell therapy can restore cochlear structure and function through hair cell regeneration, thus fundamentally restoring hearing. Stem cell therapy has been successfully studied in animal models, allowing the clinical use of endogenous and exogenous stem cells to regenerate hair cells in the inner ear of mammals, but there are still problems such as the risk of tumors after stem cell transplantation and the control of stem cell growth in specific transplant sites. The complexity of stem cell differentiation makes stem cell therapy limited, and physical stimulation such as electrical stimulation, can accurately control cells by controlling the time, intensity, and area of stimulation. Researchers combine graphene‐conductive carbon nano‐scaffolds with electric fields to promote the differentiation of NSCs.^[^ ^40^ ^]^ can control cells accurately by controlling the time, intensity, and area of stimulation, so the combination of physical stimulation and stem cell therapy has great prospects. Therefore, the combination of physical stimulation and stem cell therapy has great prospects in the treatment of SNHL.

The study of gene therapy in animal models has established its curative effect on congenital hearing loss. At the same time, some articles have proved the safety and effectiveness of AAV1‐hOTOF in the treatment of children with autosomal recessive deafness.^[^ ^220^ ^]^ This also provides a new direction for optogenetics's research on hearing recovery. This brings great hope to the treatment of sensorineural hearing loss. The researchers are trying to reconstruct the structure of AAV vectors through emerging technologies, such as artificial intelligence, to give them higher transduction efficiency, stability, and precision. Optogenetic treatment of SNHL with AAV vectors constructed in this way to carry optogenetic proteins will hopefully take the combination of photostimulation and gene therapy to a higher level.^[^ ^189^ ^]^

## 6 Summary

**Figure 17:**
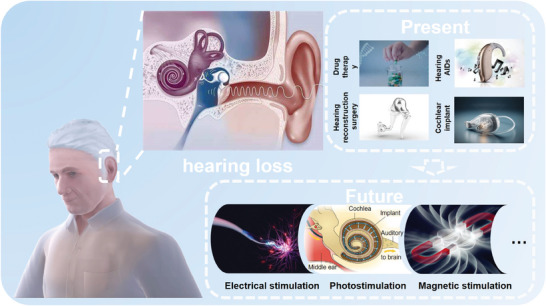
Existing measures and future directions in the field of hearing regeneration.

Degeneration of SGNs leads to severe hearing loss. The regulation of their survival and regeneration is one of the urgent medical problems. Many methods have been developed to modulate SGNs, and physical stimulations have shown great potential in the repair and regeneration of SGNs. This paper reviewed the existing hearing loss treatments and different SGNs stimulation strategies based on electricity, light, and magnetism in recent years. The mechanism of different physical stimuli on SGNs were elucidated, and the existing problems and the future research directions according to the clinical needs and practical translation were discussed (**Figure** **17**). Although physical stimulations have a broad application prospect in the field of SGNs, how to apply them widely in clinical practice still needs further exploration.

## Conflict of Interest

The authors declare no conflict of interest.
